# Flavonoid and Phenolic Acid Profiles of Dehulled and Whole *Vigna subterranea* (L.) Verdc Seeds Commonly Consumed in South Africa

**DOI:** 10.3390/molecules27165265

**Published:** 2022-08-18

**Authors:** Jane N. C. Okafor, Mervin Meyer, Marilize Le Roes-Hill, Victoria A. Jideani

**Affiliations:** 1 Food Science and Technology Department, Cape Peninsula University of Technology, Bellville 7535, South Africa; 2 Nutrition and Toxicology Division, Federal Institute of Industrial Research, Oshodi (FIIRO), P.M.B 21023, Ikeja, Lagos 100261, Nigeria; 3 Nanotechnology Innovation Centre, Department of Biotechnology, University of the Western Cape, Private Bag x17, Bellville 7535, South Africa; 4 Applied Microbial and Health Biotechnology Institute, Cape Peninsula University of Technology, Bellville 7535, South Africa

**Keywords:** Bambara groundnut landraces, bioactive molecules, characterization, flavonoid, phenolic acid, phenolic compounds, whole seed, cotyledon, quantification, mass spectrometry

## Abstract

Bambara groundnut (BGN) is an underexploited crop with a rich nutrient content and is used in traditional medicine, but limited information is available on the quantitative characterization of its flavonoids and phenolic acids. We investigated the phenolic profile of whole seeds and cotyledons of five BGN varieties consumed in South Africa using UPLC-qTOF-MS and GC-MS. Twenty-six phenolic compounds were detected/quantified in whole seeds and twenty-four in cotyledon, with six unidentified compounds. Flavonoids include flavan-3-ol (catechin, catechin hexoside-A, catechin hexoside-B), flavonol (quercetin, quercetin-3-O-glucoside, rutin, myricetin, kaempherol), hydroxybenzoic acid (4-Hydroxybenzoic, 2,6 Dimethoxybenzoic, protocatechuic, vanillic, syringic, syringaldehyde, gallic acids), hydroxycinnamic acid (trans-cinnamic, p-coumaric, caffeic, ferulic acids) and lignan (medioresinol). The predominant flavonoids were catechin/derivatives, with the highest content (78.56 mg/g) found in brown BGN. Trans-cinnamic and ferulic acids were dominant phenolic acid. Cotyledons of brown and brown-eyed BGN (317.71 and 378.59 µg/g) had the highest trans-cinnamic acid content, while red seeds had the highest ferulic acid (314.76 µg/g) content. Colored BGN had a significantly (*p* < 0.05) higher content of these components. Whole BGN contained significantly (*p* < 0.05) higher amount of flavonoids and phenolic acids, except for the trans-cinnamic acid. The rich flavonoid and phenolic acid content of BGN seeds highlights the fact that it is a good source of dietary phenolics with potential health-promoting properties.

## 1. Introduction

Numerous phytochemicals with pharmacological properties that promote health have been found in legumes. As a result, legumes/legume-based foods are gaining more interest, resulting in their increased production and utilization [1]. Among the various phytochemicals identified to date, polyphenolic compounds have attracted considerable attention because of their many pharmacological activities and health-promoting benefits [2]. Epidemiological studies have shown that persistent consumption of legumes and whole grain reduces the risk of developing chronic diseases associated with oxidative stress and damage due to their rich polyphenol composition [3,4]. Phenolic compounds are plant secondary metabolites that are distinguished from other phytochemicals by their chemical structure, which has not less than one phenol unit [5]. Depending on their various carbon skeletons varying from simple to highly complex compounds, they are grouped into subclasses, such as flavonoids, phenolic acids, tannins, stilbenes, curcuminoids, coumarins, lignans and quinones [6]. Phenolic compounds are of special interest, since they not only contribute to legume seed color and sensory properties but possess numerous biological activities that include antioxidant, anti-inflammatory, antimicrobial, antiviral, anti-atherogenic, cardioprotective, antidiabetic, antithrombotic, anticancer, neuroprotective and vasodilatory effects [7]. The major phenolic compounds present in legumes are flavonoids, phenolic acids and condensed tannins, which are normally present in a conjugated form with mono-, di- and oligosaccharides joined to one or more phenolic group, and as functional derivatives, such as esters or methyl esters [7]. Many foods, including legumes, are rich in flavonoids (flavonols, flavanals, flavanones, anthocyanidins and isoflavones) and phenolic acids (hydroxybenzoic and hydroxycinnamic acid derivatives), representing 60 and 30% of total polyphenols, respectively, in foods [7]. The health-promoting ability of phenolic compounds is associated with their antioxidant capability, which is related to their chemical structure. Normally, flavonoid compounds show more potent antioxidant activity than the non-flavonoids, and the free forms have higher activity than the combined forms, such as glycosides [8]. Similarly, hydroxycinnamic acids among the non-flavonoids demonstrate greater antioxidant activity [9]. Polyphenols disrupt chain oxidation reactions in cellular components because their phenolic groups can accept an electron to form stable phenoxy radicals. This ability to form stable phenoxy radicals is responsible for their ability to prevent various degenerative diseases, including different types of cancer [10,11], inflammation, cardiovascular disease [12], diabetes [13,14], osteoporosis, neurodegenerative disease [15], obesity, metabolic syndrome [16,17], anti-platelet aggregation, antioxidant, anti-allergic and antiglycation effects [18,19,20].

*Vigna subterranea* known as Bambara groundnut (BGN), is an underexploited crop with a rich nutrient profile and is used due to its health-promoting properties by various communities [21]. Indigenous knowledge links BGN consumption with various medicinal benefits, for example, chewing the seeds is believed to stop nausea/vomiting and morning sickness during pregnancy [22,23] and assists with the treatment of some malignancies and inflammatory disorders [24,25]. The mature seeds were reported to be used for alleviation of swollen jaw diseases in Ghana [22,26], treatment of skin rashes and sick children in Ghana [22,26], diarrhea in Ghana, Côte d’Ivoire and Kenya [22,26,27,28], impotence and in traditional medicine in Botswana [21,29], polymenorrhea, cataracts and internal bruising in Kenya and Zambia [21], venereal diseases/gonorrhea and protein malnutrition related disorder in Nigeria and Côte d’Ivoire [21,30], treatment of ulcer and to overcome kwashiorkor (protein malnutrition or edematous malnutrition) in Côte d’Ivoire/Central Africa [21]. Despite these numerous medicinal activities/health-promoting claims linked to BGN, there is limited scientific evidence to show they contain nutraceutical components with the potential to exhibit pharmacological activity.

Specific studies on BGN have shown the presence of phenolic compounds [31,32,33,34,35,36]. Similarly, several studies have demonstrated the antioxidant ability of various BGN extracts [36,37,38,39,40,41]. The health-promoting effects of phenolic compounds in legumes are linked to their potent antioxidant activity/capability, which is related to their chemical structure. Phenolic substances achieve their antioxidant properties by scavenging radical nitrogen species and radical oxygen species (RNS and ROS), inhibiting numerous enzymes and chelating trace metals taking part in the production of free radicals. This is achieved by suppression of ROS and RNS formation and increasing the rate and protection of antioxidant defense [42]. Many population-based studies have shown a positive association between the consumption of polyphenol-rich foods and a decrease in various non-communicable diseases; for example, the polyphenol in legumes and nuts decreases the risk of developing ischemic heart disease, stroke and diabetes [43], myocardial infarction and cardiometabolic health/cardiovascular disease [44,45,46,47], cognitive decline [48,49,50] and breast cancer [51].

However, the limited information or data on the detailed characterization of the phenolic compounds (phenolic acids and flavonoids) in BGN obviously and remarkably restrict the understanding of its pharmacological properties, particularly those that appear to confer health benefits that could help explain the indigenous knowledge associating BGN with medicinal value in various communities. Hence, the objective of this study was to qualitatively and quantitatively determine the distribution and quantity of phenolic compounds (flavonoids, phenolic acids, lignans and condensed tannins) in the cotyledons and whole seeds of five BGN varieties commonly produced and consumed in South Africa and provide information concerning the content and quantity of phenolic components in these BGN landraces.

## 2. Results

### 2.1. Quantitative Characterization of Phenolic Compounds in Whole Seed and Cotyledon BGN Extracts

In the present investigation, UPLC-qTOF-MS and GC-MS were utilized to qualitatively and quantitatively elucidate the phenolic compounds present in five varieties of BGN commonly consumed in South Africa. These techniques have some advantages when compared to the spectrophotometric techniques normally used for studying phenolics in legumes, in that they are very sensitive and are ideal for both separation of phenolic compounds and their quantification [52,53]. Various classes of phenolic compounds were present in the different BGN landraces studied, including flavonoids, phenolic acids, lignan and six unidentified or unknown compounds. These phenolic compounds were identified using their various retention times (R_t_) and quantified by measuring their peak area relative to that of the standard [54]. The retention times and different peak areas are shown in Figures S1–S10 (Supplementary Materials). The retention time of the compounds was as follows: catechin (10.78 min), catechin hexoside-A (8.77 min), catechin hexoside-B (10.88 min), quercetin, (22.94 min), quercetin-3-O-glucoside (17.48 min), rutin (15.94 min), myricetin (19.44 min), kaempherol (24.74 min) and medioresinol (13.03 min). Other unidentified compounds found in BGN varieties with their retention times were compounds 655 (24.15 min), 305 (7.50 min), unknown gly 421 (7.88 min), 205 (9.01 min), 381 (16.63 min) and 691 (11.24 min).

The quantitative estimates of the identified phenolics present in whole seeds and cotyledons of black, red, brown, black-eyed and brown-eyed BGN extracted with 70% (*v/v*) ethanol are presented in Table 1 and Table 2. There were fifteen phenolic compounds, which were mostly flavonoids in the flavan-3-ol and flavonol subclass. The flavan-3-ols identified were catechin, catechin hexoside-A and catechin hexoside-B, while the flavonols identified were quercetin, quercetin-3-O-glucoside, rutin, myricetin and kaempherol. Another class of phenolics also identified is medioresinol, which is a lignan.

However, six of the compounds detected were unidentified (655, 305, 421 gly, 205, 381 and 691). There were significant (*p* ≤ 0.05) differences in flavonoid content among the BGN varieties. The BGN varieties with colored seed coats (black, red and brown) had significantly (*p* ≤ 0.05) higher flavan-3-ol (catechin, catechin hexoside-A and catechin hexoside-B), quercetin-O-glucoside and rutin content than the light colored varieties (black-eyed and brown-eyed). Brown BGN had a significantly (*p* ≤ 0.05) higher concentration of flavan-3-ol: catechin (7.79 mg/g), catechin hexoside-A (78.56 mg/g) and catechin hexoside-B (12.24 mg/g) than the red and black BGN. The red variety had a significantly (*p* ≤ 0.05) higher content of quercetin-3-O-glucoside (4.52 mg/g) and rutin (7.88 mg/g) than the brown and black BGN. The black-eyed and brown-eyed BGN had significantly (*p* ≤ 0.05) lower content of these flavonoids, with the black-eyed variety having the lowest content. The black-eyed and brown-eyed varieties had significantly (*p* ≤ 0.05) higher concentrations of quercetin than the red and brown BGN, with the black-eyed having the highest concentration of 0.26 mg/g. Quercetin was not detected in the black BGN. Medioresinol was also present in the BGN landraces, and the colored seed coat varieties (black, red and brown) had significantly (*p* ≤ 0.05) higher content, with brown BGN having the highest concentration of 57.08 mg/g, while the brown-eyed variety had the lowest content of 24.07 mg/g. Kaempherol was present only in the brown variety, while myricetin was only detected in the black-eyed and brown-eye BGN, with a higher concentration of these in the black-eyed BGN. Other compounds were present in the BGN varieties that were not fully identified; they were quantified based on the peaks and tentatively labeled as compounds 655, 305, unknown 421 GLY, 205, 381 and 691, based on their retention times and peak areas.

These compounds were also significantly (*p* ≤ 0.05) higher in the black, red and brown varieties than the black-eyed and brown-eyed types, with unknown gly 421 and compound 205 of the brown BGN having the highest values of 102.25 mg/g and 111.44 mg/g, respectively.

The quantitative estimate of the identified flavonoids present in BGN cotyledons is presented in Table 2. BGN cotyledons contained similar groups of flavonoids comparable to the whole BGN, namely: catechin, catechin hexoside-A, catechin hexoside-B, quercetin, quercetin-3-O-glucoside, rutin, myricetin and lignan. Other unidentified compounds were 655, 305, 421 gly, 205, 381 and 691. However, the concentration of these compounds was significantly (*p* ≤ 0.05) lower in the BGN cotyledons compared to those of the whole seeds summarized in Table 1. Similar to the trend found in the whole seeds, the black, red and brown dehulled BGN had significantly (*p* ≤ 0.05) higher contents of catechin, catechin hexoside-A and catechin hexoside-B than the black-eyed and brown-eyed BGN. Quercetin, quercetin-3-O-glucoside and rutin were also significantly (*p* ≤ 0.05) higher in the black, red and brown dehulled BGN varieties. Quercetin and myricetin were found in the cotyledons of the black BGN variety, unlike in the whole seed. Medioresinol was also found in the dehulled BGN; the highest concentration was detected in the black BGN. Other unidentified compounds, 655, 305, 421 gly, 205, 381 and 691, were also recorded; however, their concentration was significantly (*p* ≤ 0.05) lower than those in whole BGN seeds (Table 1).

### 2.2. Quantitative Characterization of Phenolic Acids Present in Whole Seed and Cotyledon BGN Extracts

The quantitative estimates of the identified phenolic acids present in the various BGN landraces using GC-MS are presented in Table 3. The five BGN varieties (black, red, brown, black-eyed and brown-eyed) contained different classes of phenolic acids, which included hydroxybenzoic acid derivatives and hydroxycinnamic acid derivatives. The hydroxybenzoic acid/derivatives present in whole BGN varieties included 4-hydroxybenzoic acid (*p*-hydroxybenzoic acid), 2,6-dimethoxybenzoic acid, protocatechuic acid, vanillic acid, syringic acid, syringaldehyde and gallic acid, while the hydroxycinnamic acid/derivatives were *trans*-cinnamic acid, *p*-coumaric acid, caffeic acid and ferulic acid.

There were significant (*p* ≤ 0.05) differences among the BGN varieties in their phenolic acid composition. Brown and brown-eyed BGN had significantly (*p* ≤ 0.05) higher content of 4-hydroxybenzoic acid than the black, red and black-eyed types. Black-eyed BGN had the highest content of 2,6-dimethoxybenzoic acid, but it was not significantly higher than that of whole black BGN. The various BGN landraces differed significantly (*p* ≤ 0.05) in their protocatechuic and vanillic acid content. Red BGN had a significantly (*p* ≤ 0.05) higher content of protocatechuic acid than others, while the brown variety had the highest content of vanillic acid. Syringic acid was only detected in black BGN and in high amounts (216.96 µg/g). Red BGN had a significantly (*p* ≤ 0.05) higher content of syringaldehyde compared to the rest of the BGN varieties; the black-eyed BGN had the lowest content of this phenolic acid. Brown BGN had a significantly (*p* ≤ 0.05) higher content of gallic acid compared to the rest, while the black-eyed BGN had the lowest. *Trans*-cinnamic acid was the most abundant phenolic acid in the BGN varieties, and the black BGN had a significantly (*p* ≤ 0.05) higher content (232.30 µg/g) than the other varieties. Brown BGN had the lowest *trans*-cinnamic acid content (157.86 µg/g). The *p*-coumaric acid content differed significantly (*p* ≤ 0.05) among the five varieties of BGN, with the brown-eyed variety having the highest content and the black-eyed variety the lowest content. Similarly, the brown-eyed BGN had a significantly (*p* ≤ 0.05) higher caffeic acid content than the other varieties. Ferulic acid was the second most abundant phenolic acid in the BGN varieties investigated; the red variety had a significantly (*p* ≤ 0.05) higher concentration (314.76 µg/g) than the other varieties, with the black-eyed BGN having the lowest content (116.56 µg/g).

The quantitative estimates of the identified phenolic acid content of the BGN cotyledons are presented in Table 4. BGN cotyledons contain similar classes of phenolic acids as the whole seed. However, these were significantly (*p* ≤ 0.05) lower than those in the whole seed, except for the trans-cinnamic acid, which was higher in the cotyledon of the black, red, brown and brown-eyed BGN varieties. Syringic acid was not detected in any of the dehulled BGN cotyledons.

Black-eyed BGN had a significantly (*p* ≤ 0.05) higher 4-hydroxybenzoic acid content than the black, red, brown and brown-eyed BGN. The brown BGN cotyledons had the highest content (26.01 µg/g) of 2,6-dimethoxybenzoic acid, but it was not significantly different to the others. The brown BGN cotyledons also had the highest protocatechuic acid (26.28 µg/g) content, which was significantly (*p* ≤ 0.05) higher than for the other varieties. The cotyledons from the red variety had the highest concentration of vanillic acid (50.63 µg/g) and syringaldehyde content (153.50 µg/g), which were significantly (*p* ≤ 0.05) higher than the black, brown, black-eyed and brown-eyed varieties. The brown BGN cotyledons had a significantly (*p* ≤ 0.05) higher concentration of gallic acid (41.59 µg/g) than that of the other varieties. The dehulled BGN cotyledons had a relatively high concentration of *trans*-cinnamic acid, and this was significantly (*p* ≤ 0.05) highest in brown-eyed (378.59 µg/g) and also higher than those in whole BGN seeds, while the red dehulled BGN had the lowest content (261.54 µg/g). Dehulled red BGN had significantly (*p* ≤ 0.05) higher concentrations of *p*-coumaric acid and ferulic acid than the other BGN varieties (74.49 µg/g and 227.32 µg/g, respectively). Black BGN cotyledons had a significantly (*p* ≤ 0.05) higher concentration of caffeic acid, while the brown-eyed BGN had the lowest concentration (58.93 µg/g).

### 2.3. Bambara Groundnut Seed Clusters in Relation to Flavonoid and Phenolic Acid Content

**Figure 1:**
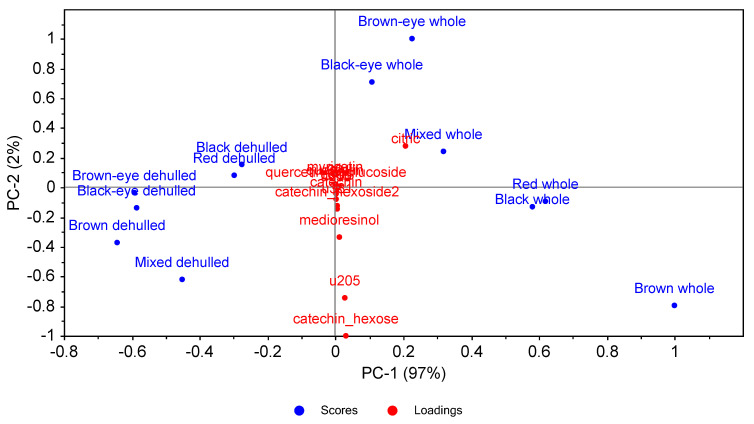
Principal component scores and loadings based on Bambara groundnut form and variety with regard to flavonoids and lignan composition/characterization.

**Figure 2:**
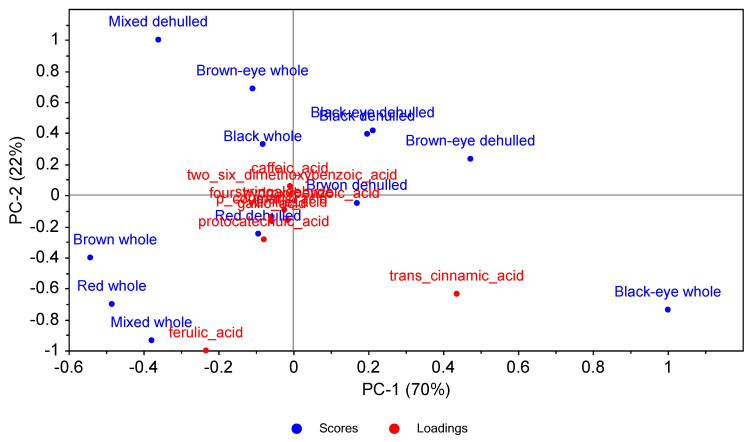
Principal component scores and loadings based on Bambara groundnut form and variety with regard to phenolic acid composition.

Figure 1 details the relationship between the BGN landraces and their flavonoid content. The first component explains 97% of the variation and the second component 2%. Similarly, two principal components explain the variation in phenolic acid of BGN landraces in Figure 2. The first component explains 70% of the variation and the second 22%.

## 3. Discussion

### 3.1. Flavonoids and Lignan in Whole BGN Seeds and Cotyledons

The present investigation shows that catechin and its derivatives, catechin hexoside-A and catechin hexoside B, predominate in all the BGN varieties, with colored varieties having the highest concentration, which was also significantly (*p* ≤ 0.05) higher in the whole seeds compared to the cotyledons (dehulled BGN). Previous studies have shown that legumes with darkly pigmented seed coats, such as black, red and brown color, have a higher content of total phenolics and condensed tannins compared to less colored ones, such as the white and yellow cultivars [55,56]. Similarly, it has been demonstrated that the seed coat of legumes contains a higher concentration of phenolic compounds compared to the cotyledon and testa [57]. Previous studies reported the presence of catechin and its derivatives in BGN landraces [31,32,33,34,35]. The concentration of catechin obtained in this study was comparable to the 2.34 mg/g reported by Ref [32] but higher than the 0.30 µg/g reported by Adebiyi et al. [35] and that of Harris et al. [33]. The variation could probably be due to different extraction methods, the BGN cultivar used and differences in the environmental conditions under which the BGN was cultivated. Flavan-3-ol was the dominant flavonoid with the highest concentration in the five BGN varieties, and these compounds have been shown to possess various pharmacological properties [58]. Flavan-3-ols have demonstrated biological properties/activities and health-promoting beneficial effects, which include cardio-protective effects and prevention of platelet aggregation [59], antioxidant, anti-diabetes [60], hypolipidemic potential, antiviral activity [61], regulation of plasma LDL and HDL cholesterol [62], blood pressure [63], anti-cancer [64], neuroprotective and anti-aging effects [65]. There has been renewed interest in proanthocyanidin or condensed tannins, polymer chains of flavan-3-ols or catechins because of their strong antioxidant activity, which has been demonstrated to be higher than that of other antioxidants, such as vitamin C, and they are reported to have a positive effect on blood flow and blood pressure [63].

Studies have reported various flavonols in BGN landraces [31,32,33,34,35]. However, the quantity of quercetin obtained in this experiment was higher than the 0.070 mg/g reported by Harris et al. [33] and Adebiyi et al. [35] but lower than the 6.39 mg/g reported by Salawu [32]. The variation in concentration could be due to the different extraction solvent or time of extraction. The higher quercetin content by Salawu [32] could probably be due to the acidified methanol (lower pH) used, which might have enhanced the extractability of the phenolic compounds, while 70% (*v/v*) ethanol was used in this study. The other flavonols identified and quantified were rutin, myricetin and kaempherol, although the latter was only detected in whole brown BGN seeds. Flavonols are known as biologically active components with great health benefits and have been shown to exhibit antioxidant, antibacterial, antiviral, anticancer and cardioprotective properties [66]. Quercetin, which is often referred to as a model flavonol, has gained increasing research attention because it exhibits potent antioxidant properties [67], strong potential in the treatment of cancers [68], anti-bacterial activity, anti-viral properties [69], anti-obesity [70] and anti-inflammatory activities [71] and ability to prevent cardiovascular diseases [72]. Quercetin intake (730 mg/day for four weeks) by hypertensive patients was shown to reduce systolic pressure (by 7 mm Hg), diastolic pressure and mean arterial pressure [73]. Another interesting compound found in both whole BGN seeds and cotyledons is medioresinol but at higher concentrations compared to the 0.80 μg/g reported by Adebiyi et al. [35]. The difference in data may probably be due to the different maturity/harvesting stages of BGN and variation in the cultivars used in the study. Medioresinol belongs to the lignan subgroup of polyphenols and has a chemical structure that is similar to steroids, specifically phytoestrogen. Lignans have received increasing research attention because of their numerous health-promoting benefits that include reduced risk of osteoporosis, heart or cardiovascular disease, different types of cancers, particularly breast cancer, menopausal symptoms, as well as antioxidant and anti-inflammatory activity [74]. The perceived significantly higher content of flavonoids in whole BGN seeds compared to the cotyledon (dehulled seeds) was due to the seed coats in the whole seeds. The seed coat of legumes has been shown to contain a high concentration of phenolic compounds. Legume seed coats could be a rich source of polyphenols and natural antioxidants [75]. Therefore, processing treatments, such as dehulling, result in the removal of the phenolic compounds, thereby reducing the overall polyphenol content of processed legumes, which includes BGN [76].

### 3.2. Phenolic Acids Present in Whole BGN Seeds and Cotyledons

To further explore the nutraceutical potential of whole BGN seeds and cotyledons, the presence and concentrations of phenolic acids in the different BGN landraces were analyzed and quantified. The finding indicated the presence of eleven phenolic acids, which were grouped into hydroxybenzoic acid derivatives and hydroxycinnamic acid derivatives. The seven hydroxybenzoic acids identified in both whole BGN seeds and cotyledons of the various landraces were 4-hydroxybenzoic acid (also known as *p*-hydroxybenzoic acid), protocatechuic acid, 2,6-dimethoxybenzoic acid, vanillic acid, syringaldehyde, syringic acid and gallic acid, while the four hydroxycinnamic acid/derivatives detected were *trans*-cinnamic acid, *p*-coumaric acid, caffeic acid and ferulic acid. Whole BGN seeds of the different varieties had significantly higher concentrations of almost all phenolic acids, except the *trans*-cinnamic acid (cinnamic acid), which was significantly higher in the cotyledons of black, red, brown and brown-eyed BGN varieties. Syringic acid was found in relatively high concentrations only in the whole seed of the black BGN variety. Hydroxycinnamic acid was the predominant phenolic acid in BGN, with *trans*-cinnamic acid and ferulic acid being the most prevalent or abundant in both whole BGN seeds and cotyledons of the various BGN landraces tested. Among the phenolic acids identified/quantified in this study, only gallic acid, caffeic acid/derivatives, *p*-coumaric acid and syringic acid/derivatives have been previously identified in whole BGN [31,32,33,34,35]. However, the concentration of gallic acid obtained in the present study was significantly higher than the 0.005 mg/g reported for red whole BGN seeds by Harris et al. [33] but lower than the 1.03 mg/g for gallic acid and 3.65 mg for caffeic acid obtained by Salawu [32]. The variation in the content of these phenolic acids with previous studies could probably be due to the difference in the extraction solvent, extraction time, variety of BGN and other environmental factors. Extraction time and the solvent used in extractions are contributory factors that affect the extractability of phenolic compounds, and a longer period of interaction with the solvent enhances better extraction yield [77]. In addition, genetic factors, such as variety and environmental conditions, processing and storage, will affect the phenolic composition. Previous research has identified vanillic acid, syringaldehyde, syringic acid and ferulic acid in various varieties of the common bean [78]. Protocatechuic acid has been reported in seeds of field peas, beans, lentils and varieties of almond and *Vicia sativa* seeds [79,80]. *Trans*-cinnamic acid was detected in Betchuana white cowpea [81], germinated black beans, mung beans, peanuts, Adzuki beans, soybeans and white cowpea seeds [82]. 4-Hydroxybenzoic acid has been found in peas, beans, lentils, *Vigna sativa* seeds and *Lamiaceae* spices [79,80].

Phenolic acids amid all other phenolic compounds have displayed exceptional health benefits [20,83,84,85]. The phenolic acids quantified in the whole BGN seeds and cotyledons in this study are of considerable interest because of their pharmacological properties. Of special interest and worthy of mentioning are some biological activity and health-promoting properties of the important ones; for instance, gallic acid has been shown to exhibit antioxidant, cardioprotective and anti-inflammatory properties [86], anti-hypertension effects by preventing the activity of angiotensin-converting enzymes and reducing blood pressure in hypertensive rats [87], anti-obesity properties [88] and anti-cancer potentials [89]. A consistent single intake of 15 mg/kg of gallic acid extract for 7 days reduced oxidative damage and inflammatory biomarker CRP in diabetic patients [90]. Vanillic acid possesses antioxidant, cardioprotective, anti-inflammatory and neuroprotective effects [91]. Caffeic acid was reported to exert anti-hyperglycemia, anti-hyperlipidemia, anti-inflammatory effects [92] and positively controlled blood pressure in hypertensive rats [93] and anti-cancer/tumor potentials [94]. A daily consumption of a 0.3 g caffeic acid tablet (three times per day) for 12 weeks increased the platelet count in primary immune thrombocytopenia patients [95]. Ferulic acid and *trans*-cinnamic acid (the predominant phenolic acids in these BGN varieties) were reported to exhibit vast pharmacological activities, such as antioxidant, cardioprotective, anti-inflammatory, antithrombotic, hepatoprotective [20,96,97,98], anti-ageing and anti-hypertensive effects [99], anti-carcinogenic properties, activation of transcriptional factors, gene expression and signal transduction, inhibition of tumor development, cell-cycle arrest and reduction in metastatic nodule formation [83,100], anti-diabetic and antihypertensive effects by lessening/relieving insulin resistance and hypertension and restoration of NO levels linked with metabolic syndrome [101,102], antiallergic, antimicrobial and antiviral activities, and increase sperm viability [20]. The administration of 500 mg (twice daily) ferulic acid capsule to hyperlipidemic patients for six weeks significantly decreased an oxidative stress biomarker (MDA), oxidized LDL and inflammatory markers and improved their lipid profile [103]. The amazing biological activities of some of these compounds present in BGN varieties could probably partly explain the traditional medicinal use of BGN in various parts of Africa and other parts of the world for the treatment or prevention of various diseases.

### 3.3. Bambara Groundnut Seed Clusters in Relation to Flavonoid and Phenolic Acid Content

Figure 1 details the relationship between the BGN landraces and their flavonoid content. The first component explains 97% of the variation and the second component 2%. The first component had large positive association with whole BGN with pigmented testa (brown, black and red), which are high in catechin hexose, u205 and medioresinol. Whole red and black BGN were similar in flavonoid content. The second component correlates with whole BGN with light colored testa (brown-eyed and black-eyed). The BGN seeds with light colored testa were negatively correlated with the dehulled light pigmented seeds and the dehulled brown BGN seeds in flavonoid content. The whole pigmented BGN seeds were negatively correlated with the dehulled pigmented seeds in flavonoid content. The flavonoids that differentiated the BGN landraces were catechin hexose, u205 and medioresinol. 

Two principal components explain the variation in phenolic acid of BGN landraces (Figure 2). The first component explains 70% of the variation and the second 22%. The first component separates the whole black-eyed BGN seeds that are high in trans-cinnamic acid from the whole pigmented seeds that are low in ferulic acid. The whole brown-eyed and black seeds have an average phenolic acid content. The dehulled seeds have similar phenolic acid contents. The whole seeds are negatively correlated with the dehulled seeds in phenolic acid content. The whole brown-eyed and black seeds are negatively correlated with the whole black-eyed seeds in terms of their phenolic acid content.

## 4. Materials and Methods

### 4.1. Sample Collection

**Scheme 1:**
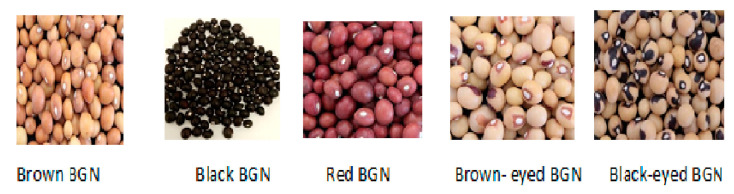
Picture of different varieties of Bambara groundnut (BGN) studied.

Bambara groundnut seeds were grown by a farmer in Nguthuthu area in the KwaZulu-Natal Province of South Africa, between October and early December 2016, and were harvested in June 2017 using a groundnut harvester. The pods were shelled with modified groundnut sheller and packaged in sealed containers and transported to the Cape Peninsula University of Technology (CPUT) for the research study. The seeds were sorted into black, red, brown, brown-eyed and black-eyed BGN, according to the testa colors, and kept in airtight plastic zip lock bags until further analysis (Scheme 1).

### 4.2. Preparation of BGN Seeds and Cotyledons

The black, red, brown, brown-eyed and black-eyed BGN seeds were washed with distilled water and dried at 50 °C using an industrial hot air drier (Geiger and Klotzebucher, Cape Town, South Africa) for 48 h. Whole BGN seeds (black, red, brown, brown-eyed and black-eyed) were separately milled into flour (250 mm sieve size) using a hammer mill (PertenMill, Perten Instruments AB, Sweden) and packaged in airtight plastic zip lock bags. Bambara groundnut cotyledons were produced by first coarse milling of the BGN seeds (black, red, brown, brown-eyed and black-eyed BGN) with a Corona manual grain crusher for easier manual removal of the seed coat to produce the BGN cotyledons. The cotyledons were milled into flour (250 mm sieve size) using a PertenMill (Perten Instruments AB, Sweden) and packaged in airtight plastic zip lock bags. The BGN flours from black, red, brown, brown-eyed and black-eyed BGN were stored at 4–6 °C until required for further analysis.

### 4.3. Preparation of BGN Extracts

The milled Bambara groundnut whole seeds and cotyledons from each variety were extracted by placing 50 g of the sample in 500 mL 70% (*v/v*) ethanol solution and allowed to extract over 72 h by maceration at 25 °C with constant shaking in a mechanical shaker [104,105]. Following extraction, the mixture was centrifuged at a speed of 15,316× *g* for 15 min at 4 °C (Beckman Coulter Avanti J-E centrifuge, USA) and filtered through Whatman filter paper (0.45 µm pore size). The supernatant obtained was concentrated to approximately 30 mL by evaporation under pressure using a rotary evaporator (Buchi RE 011 model, Switzerland) at 40 °C to remove residual ethanol. The various BGN extracts were frozen at −80 °C and freeze dried to obtain a lyophilized powder (Bench Top Pro Omnitronics freeze dryer, SP Scientific, Warminster, PA, USA). The resulting freeze-dried extracts were refrigerated at 4 °C until further analysis.

### 4.4. Qualitative and Quantitative Polyphenol Profiling of Whole BGN Seeds and Cotyledons Using UPLC-qTOF-MS

Flavonoids and other metabolites in the extracts of whole BGN seeds and cotyledons were identified and quantified by ultra-performance liquid chromatography–mass spectroscopy (UPLC-qTOF-MS), following the method described by Stander et al. [106]. A Waters Synapt G2 quadrupole time-of-flight (qTOF) mass spectrometer was used for (UPLC-MS) analysis. It was fitted with a Waters Ultra pressure liquid chromatography (UPLC; Waters, Milford, MA, USA) used for high-resolution UPLC-MS analysis and photodiode array detection, thus allowing simultaneous collection of UV and MS spectra. Electrospray ionization was used in negative mode with a voltage of 15 V, desolvation temperature of 275 °C, desolvation gas at 650 L/h and the rest of the mass spectrometer settings optimized for best resolution and sensitivity. Separation was achieved on a Waters BEH C18, 2.1 × 100 mm column with 1.7 μm particles. A gradient was applied using 0.1% (*v/v*) formic acid (solvent A) and acetonitrile containing 0.1% (*v/v*) formic acid (solvent B). The gradient started at 100% solvent A for 1 min and changed to 28% B over 22 min in a linear way. It then proceeded to 40% B over 50 s and a wash step of 1.5 min at 100% B, followed by re-equilibration to initial conditions for 4 min. The flow rate was 0.3 mL/min, and the column was kept at 55 °C. The injection volume was 2 µL. Data were acquired in MSE mode, which consisted of a low collision energy scan (6V) from *m/z* 150 to 1500 and a high collision energy scan from *m/z* 40 to 1500. The high collision energy scan was performed using a collision energy ramp of 30–60 V. The photodiode array detector was set to scan from 220 to 600 nm. The mass spectrometer was optimized for best sensitivity, a cone voltage of 15 V, desolvation gas was nitrogen at 650 L/h and desolvation temperature, 275 °C. The instrument was operated with an electrospray ionization probe in the negative mode. Sodium formate was used for calibration, and leucine encephalin was infused in the background as a lock mass for accurate mass determinations.

Flavonoids and other metabolites were quantified relatively against a calibration curve established by injecting catechin standard in the range of 0.5–100 mg/L. Data were processed using MSDIAL and MSFINDER (RIKEN Center for Sustainable Resource Science: Metabolome Informatics Research Team, Kanagawa, Japan).

### 4.5. Phenolic Acid Analysis with GC-MS and Chromatographic Separation

The phenolic acids present in the whole BGN seed and cotyledon extracts were identified and quantified using GC-MS. Approximately 10 mg of the sample was extracted with 1000 μL 70% ethanol/water (*v/v*). An aliquot (100 μL) of naphthol at a final concentration of 100 ppb was added as internal standard. The mixture was briefly vortexed and subsequently incubated for 180 min at 60 °C. The sample was centrifuged, and the resultant supernatant was transferred into clean microcentrifuge tubes. An aliquot (500 µL) of the supernatant was then dried in a speed vac. The dry samples were reconstituted with 100 µL acetonitrile followed by 50 µL BSTFA with 1% TCMS. The mixture was vortexed and then derivatized by incubation for 1 h in an oven maintained at 80 °C. After incubation, the mixture was again vortexed and then transferred into a GC vial with an insert. An aliquot (1 µL) of the sample was injected into a Thermo TSQ 8000 MS quadrupole operated in the SRM mode.

Separation of the components of the phenolic acid was performed in a Thermo Scientific TRACETM 1310 gas chromatography coupled with a non-polar Factor Four VF-17ms (30 m, 0.25 mm ID, 0.25 µm film thickness), part number CP8982. The initial oven temperature was 100 °C, held for 4 min, and increased at 6 °C/min to 180 °C, held for 2 min with a final temperature of 250 °C at a rate of 15 °C/min, and a final hold time of 5 min. The injector and transfer line temperatures were maintained at 250 °C and 280 °C, respectively. Helium at 1 mL/min flow rate was used as a carrier gas. The ionization source temperature was set at 250 °C, and an emission current of 50 µA was used with Argon collision. Phenolic acids were quantified relatively against a calibration curve established by injecting authentic standards.

### 4.6. Statistical Analysis 

Data were presented as mean ± standard deviation of three independent replicates. Statistical analysis was conducted using the IBM Statistical Package for the Social Science (IBM SPSS, version 24). Multivariate analysis of variance (MANOVA) was used to establish the mean difference between treatments. Duncan multiple range test was used for mean separation, and a significant difference was determined at *p* ≤ 0.05. The principal component analysis was employed to draw out the component that described the variability in the standardized data using cross-validation with singular value decomposition (SVD) algorithm (UnscramblerX 10.4, 2016).

## 5. Conclusions

Twenty-six phenolic compounds from the flavan-3-ol, flavonol, lignan, hydrobenzoic acid and hydrocinnamic acid subclass of phenolics were detected in BGN landraces. Catechin/derivatives (flvan-3-ol) were the predominant flavonoid, while *trans*-cinnamic acid and ferulic acid/derivatives (hydroxycinnamic acid) were the most abundant phenolic acids present in the BGN landraces, with the colored varieties having a higher content. Whole BGN seeds of different landraces had higher concentrations of flavonoids and phenolic acids, except for the *trans*-cinnamic acid, which was higher in the BGN cotyledon extracts. The results from this investigation provide valuable information and documentation on the phenolic compound profile (flavonoids, lignan and phenolic acids) of whole and dehulled seeds (cotyledons) of BGN landraces commonly consumed in South Africa. The numerous biologically active phenolic compounds present in the BGN landraces portray them as a source of health-promoting food because the pharmacological properties of these phenolic compounds are well established and could be explored as an important option for healthy nourishments. However, additional research is needed to test the pharmacological activity of the identified flavonoids and phenolic acids in a biological system to verify the contribution under specific health conditions.

## Figures and Tables

**Table 1:** Flavonoids detected in ethanolic extracts of the whole seeds of five different Bambara groundnut landraces.

	Bambara Groundnut Landrace ^1^				
Flavonoids (mg/g)	Black	Red	Brown	Black-Eyed	Brown-Eyed
Catechin hexoside A	60.44 ± 1.94 ^a^	53.26 ± 1.48 ^b^	78.56 ± 0.99 ^c^	3.67 ± 0.90 ^d^	4.12 ± 0.41 ^d^
Catechin hexoside B	7.13 ± 0.81 ^a^	6.29 ± 0.58 b ^a^	12.24 ± 1.09 ^d^	0.41 ± 0.11 ^c^	0.52 ± 0.03 ^c^
Catechin	3.00 ± 1.69 ^ab^	4.09 ± 0.65 ^a^	7.79 ± 0.27 ^b^	0.74 ± 0.13 ^c^	0.82 ± 0.12 ^c^
Quercetin-3-O-glucoside	1.21 ± 0.24 ^a^	4.52 ± 0.15 ^b^	1.11 ± 0.66 ^a^	0.53 ± 0.09 ^c^	0.64 ± 0.04 ^c^
Quercetin	ND	0.08 ± 0.15 ^a^	0.17 ± 0.14 ^b^	0.23 ± 0.02 ^bc^	0.26 ± 0.03 ^c^
Rutin	1.02 ± 0.01 ^a^	7.88 ± 0.74 ^b^	1.37 ± 0.20 ^a^	0.48 ± 0.12 ^c^	0.51 ± 0.06 ^c^
Myricetin	ND	ND	ND	1.58 ± 0.12 ^a^	1.35 ± 0.12 ^b^
Kaempferol	ND	ND	0.057	ND	ND
Medioresinol	30.51 ± 0.99 ^a^	33.95 ± 2.65 ^a^	57.08 ± 4.25 ^b^	24.86 ± 1.52 ^c^	24.07 ± 0.80 ^c^
655	9.96 ± 0.80 ^a^	10.31 ± 0.70 ^a^	10.93 ± 2.71 ^a^	6.79 ± 0.81 ^b^	6.92 ± 0.36 ^b^
305	3.32 ± 0.38 ^a^	3.21 ± 0.328 ^a^	6.02 ± 0.23 ^b^	2.50 ± 0.17 ^c^	2.93 ± 0.25 ^c^
Unknown 421 GLY	89.78 ± 2.44 ^a^	97.11 ± 3.44 ^b^	102.25 ± 3.08 ^d^	78.91 ± 5.21 ^c^	90.54 ± 4.86 ^a^
205	67.72 ± 5.95 ^a^	75.27 ± 2.81 ^a^	111.44 ± 7.10 ^b^	44.55 ± 4.22 ^c^	35.03 ± 2.67 ^d^
381	12.67 ± 9.38 ^a^	20.18 ± 1.05 ^a^	36.12 ± 2.70 ^b^	19.06 ± 1.50 ^a^	18.51 ± 0.25 ^a^
691	0.53 ± 0.29 ^ab^	0.25 ± 0.10 ^a^	1.06 ± 0.38 ^c^	0.40 ± 0.07 ^ab^	0.75 ± 0.07 ^bc^

^1^ Values are mean ± standard deviation of three replicates. Means in the same row followed by different superscript letters are significantly (*p* ≤ 0.05) different. ND—Not detected.

**Table 2:** Flavonoids detected in the ethanolic extracts of the cotyledons of five different Bambara groundnut landraces.

	Bambara Groundnut Landrace ^1^				
Flavonoids (mg/g)	Black	Red	Brown	Black-Eyed	Brown-Eyed
Catechin hexoside-A	7.45 ± 0.45 ^a^	6.76 ± 0.96 ^a^	17.36 ± 0.85 ^b^	2.78 ± 0.32 ^c^	1.27 ± 0.17 ^c^
Catechin hexoside-B	1.03 ± 0.25 ^a^	2.59 ± 1.12 ^b^	2.66 ± 0.49 ^b^	0.30 ± 0.05 ^c^	0.17 ± 0.03 ^c^
Catechin	0.77 ± 0.21 ^a^	0.69 ± 0.10 ^a^	1.47 ± 0.16 ^b^	0.34 ± 0.10 ^c^	0.27 ± 0.16 ^c^
Quercetin-3-O-glucoside	0.58 ± 0.05 _ab_	0.70 ± 0.07 ^b^	0.38 ± 0.04 ^a^	0.52 ± 0.08 ^ab^	0.58 ± 0.08 ^ab^
Quercetin	0.27 ± 0.03 ^a^	0.27 ± 0.035 ^a^	0.17 ± 0.01b ^a^	0.25 ± 0.01 ^ab^	0.11 ± 0.012 ^b^
Rutin	0.22 ± 0.08 ^a^	0.68 ± 0.08 ^b^	0.28 ± 0.01 ^a^	0.42 ± 0.08 ^d^	0.05 ± 0.03 ^c^
Myricetin	0.89 ± 0.04 ^a^	ND	ND	1.65 ± 0.07 ^b^	1.36 ± 0.10 ^c^
Kaempherol	ND	ND	ND	ND	ND
Medioresinol	28.04 ± 1.05 ^a^	25.26 ± 2.90 ^bc^	21.58 ± 0.96 ^d^	23.32 ± 0.72 ^b^	23.44 ± 0.8 ^b^
655	7.15 ± 0.18 ^a^	7.06 ± 0.37 ^a^	5.54 ± 0.78 ^b^	6.04 ± 0.62 ^ab^	6.38 ± 0.76 ^ab^
305	2.44 ± 0.15 ^a^	2.08 ± 0.15 ^a^	1.59 ± 0.41 ^b^	2.51 ± 0.34 ^a^	2.07 ± 0.41 ^a^
Unknown 421 GLY	86.69 ± 1.076 ^a^	94.09 ± 4.22 ^b^	64.40 ± 4.70 ^c^	71.72 ± 1.16 ^d^	79.17 ± 3.01 ^e^
205	40.53 ± 2.21 ^a^	45.20 ± 1.97 ^b^	34.03 ± 3.76 ^c^	41.11 ± 1.46 ^a^	34.26 ± 1.97 ^c^
381	10.68 ± 0.47 ^a^	20.37 ± 0.99 ^c^	11.97 ± 1.46 ^a^	18.59 ± 1.40 ^b^	18.22 ± 0.61 ^b^
691	0.28 ± 0.01 ^ab^	0.24 ± 0.05 ^a^	0.10 ± 0.08 ^a^	0.36 ± 0.07 ^b^	0.80 ± 0.12 ^d^

^1^ Values are mean ± standard deviation of three replicates. Means in the same row followed by different superscript letters are significantly (*p* ≤ 0.05) different. ND—Not detected.

**Table 3:** Phenolic acid composition of ethanolic extracts prepared from the whole seeds of various Bambara groundnut landraces.

	Bambara Groundnut Landrace ^1^				
Phenolic Acid (µg/g)	Black	Red	Brown	Black-Eyed	Brown-Eyed
4-Hydroxybenzoic acid	41.07 ± 2.38 ^a^	41.32 ± 1.37 ^a^	58.87 ± 2.43 ^d^	51.31 ± 2.25 ^c^	57.79 ± 3.64 ^d^
2,6-Dimethoxybenzoic acid	25.84 ± 2.64 ^a^	26.83 ± 2.60 ^ab^	26.77 ± 1.80 ^a^	25.19 ± 0.39 ^a^	28.94 ± 2.58 ^b^
Protocatechuic acid	66.53 ± 1.92 ^a^	77.21 ± 1.38 ^b^	71.54 ± 1.37 ^b^	15.09 ± 0.25 ^c^	20.74 ± 0.94 ^d^
Vanillic acid	39.82 ± 4.39 ^a^	51.78 ± 2.56 ^b^	56.77 ± 1.42 ^c^	45.37 ± 4.48 ^d^	31.78 ± 2.07 ^e^
Syringic acid	216.96	ND	ND	ND	ND
Syringaldehyde	61.12 ± 3.39 ^a^	190.74 ± 1.51 ^b^	61.54 ± 1.89 ^a^	53.61 ± 1.89 ^c^	74.79 ± 12.52 ^d^
Gallic acid	69.93 ± 0.35 ^a^	78.01 ± 2.48 ^b^	96.73 ± 2.82 ^c^	40.85 ± 2.24 ^d^	53.90 ± 1.49 ^e^
*Trans*-cinnamic acid	232.30 ± 56.40 ^a^	191.99 ± 30.85 ^a^	157.86 ± 24.1 ^b^	172.55 ± 25.9 ^b^	205.16 ± 62.97 ^a^
*p*-coumaric acid	99.85 ± 9.49 ^a^	121.03 ± 6.12 ^b^	106.46 ± 15.22 ^c^	55.37 ± 5.17 ^d^	124.11 ± 15.18 ^b^
Caffeic acid	67.25 ± 1.42 ^a^	63.06 ± 1.36 ^b^	63.93 ± 1.05 ^b^	58.49 ± 0.30 ^c^	103.02 ± 3.81 ^d^
Ferulic acid	144.66 ± 11.61 ^a^	314.7 ± 16.77 ^b c^	293.01 ± 22.84 ^c^	116.56 ± 1.02 ^d^	121.73 ± 1.04 ^e^

^1^ Values are mean ± standard deviation of three replicates. Means in the same row followed by different superscript letters are significantly (*p* ≤ 0.05) different. ND—Not detected.

**Table 4:** Phenolic acid composition of the ethanolic extracts of the cotyledons of various Bambara groundnut landraces.

	Bambara Groundnut Landrace ^1^				
Phenolic Acid (µg/g)	Black	Red	Brown	Black-Eyed	Brown-Eyed
4-Hydroxybenzoic acid	38.60 ± 2.74 ^a^	37.70 ± 0.38 ^a^	35.57 ± 1.07 ^b^	47.34 ± 2.04 ^c^	41.77 ± 2.05 ^d^
2,6-Dimethoxybenzoic acid	23.49 ± 2.58 ^a^	21.58 ± 2.63 ^a^	26.01 ± 1.69 ^a^	23.56 ± 5.89 ^a^	22.52 ± 2.61 ^a^
Protocatechuic acid	12.36 ± 1.55 ^a^	13.99 ± 5.97 ^ac^	26.28 ± 0.49 ^b^	14.54 ± 0.59 ^c^	6.58 ± 0.22 ^d^
Vanillic acid	35.31 ± 0.51 ^a^	50.63 ± 8.58 ^b^	29.44 ± 4.08 ^c^	43.96 ± 6.83 ^b^	27.46 ± 4.24 ^c^
Syringic acid	ND	ND	ND	ND	ND
Syringaldehyde	49.07 ± 0.97 ^a^	153.50 ± 1.72 ^d^	50.24 ± 1.29 ^a^	68.89 ± 0.68 ^c^	49.16 ± 1.01 ^a^
Gallic acid	35.82 ± 1.93 ^a^	39.67 ± 0.34 ^bc^	41.59 ± 0.40 ^c^	37.28 ± 2.58 ^ab^	35.52 ± 2.86 ^a^
*Trans*-cinnamic acid	297.3 ± 15.58 ^a^	261.54 ± 39.76 ^a^	317.71 ± 56.68 ^bc^	299.23 ± 4.81 ^a^	378.6 ± 19.92 ^d^
*p*-coumaric acid	69.27 ± 4.51 ^a^	74.49 ± 2.45 ^b^	60.24 ± 5.21 ^d^	53.37 ± 8.39 ^d c^	73.00 ± 7.05 ^a b^
Caffeic acid	64.44 ± 2.62 ^a c^	60.16 ± 0.65 ^b^	59.08 ± 0.18 ^d^	62.06 ± 1.02 ^c^	58.93 ± 1.00 ^d^
Ferulic acid	121.61 ± 4.03 ^a^	227.3 ± 12.65 ^b^	172.98 ± 3.98 ^c^	114.9 ± 22.57 ^d^	96.14 ± 3.75 ^e^

^1^ Values are mean ± standard deviation of three replicates. Means in the same row followed by different superscript letters are significantly (*p* ≤ 0.05) different. ND—Not detected.

## Data Availability

Data are contained within the article or the Supplementary Material.

## Supplementary Materials

The following supporting information can be downloaded at: https://www.mdpi.com/article/10.3390/molecules27165265/s1. Figure S1: Chromatogram of phytochemicals and mass spectra of identified flavonoids in whole black Bambara groundnut variety; Figure S2: Chromatogram of phytochemicals and mass spectra of identified flavonoids in whole red Bambara groundnut variety; Figure S3: Chromatogram of phytochemicals and mass spectra of identified flavonoids in whole brown Bambara groundnut variety; Figure S4: Chromatogram of phytochemicals and mass spectra of identified flavonoids in whole black-eyed Bambara groundnut variety; Figure S5: Chromatogram of phytochemicals and mass spectra of identified flavonoids in whole brown-eyed Bambara groundnut variety; Figure S6: Chromatogram of phytochemicals and mass spectra of identified flavonoids in the cotyledon of black Bambara groundnut variety; Figure S7: Chromatogram of phytochemicals and mass spectra of identified flavonoids in the cotyledon of red Bambara groundnut variety; Figure S8: Chromatogram of phytochemicals and mass spectra of identified flavonoids in the cotyledon of brown Bambara groundnut variety; Figure S9: Chromatogram of phytochemicals and mass spectra of identified flavonoids in the cotyledon of black-eyed Bambara groundnut variety; Figure S10: Chromatogram of phytochemicals and mass spectra of identified flavonoids in the cotyledon of brown-eyed Bambara groundnut variety; Figure S11: Chromatogram of phenolic acid and mass spectra of identified 4-Hydroxybenzoic acid Bambara groundnut varieties; Figure S12: Chromatogram of phenolic acid and mass spectra of identified trans-cinnamic acid Bambara groundnut varieties; Figure S13: Chromatogram of phenolic acid and mass spectra of identified vanillic acid in Bambara groundnut varieties; Figure S14: Chromatogram of phenolic acid and mass spectra of identified gallic acid in Bambara groundnut varieties; Figure S15: Chromatogram of phenolic acid and mass spectra of identified p-coumaric acid in Bambara groundnut varieties; Figure S16: Chromatogram of phenolic acid and mass spectra of identified syringic acid Bambara in groundnut varieties; Figure S17: Chromatogram of phenolic acid and mass spectra of identified ferulic acid in Bambara groundnut varieties.
