# Supplementary material for: Flavonoid and Phenolic Acid Profiles of Dehulled and Whole Vigna subterranea (L.) Verdc Seeds Commonly Consumed in South Africa
Source: Molecules. 2022 Aug 18;27(16):5265. doi: 10.3390/molecules27165265 (PMC9415687; doi:10.3390/molecules27165265)
Supplement: Supplementary file 1 [file molecules-27-05265-s001.zip › molecules-1799012-supplementary.pdf]

Supplementary Materials

Flavonoid and Phenolic Acid Profiles of Dehulled and Whole *Vigna subterranea* (L.) Verdc Seeds Commonly Consumed in South Africa

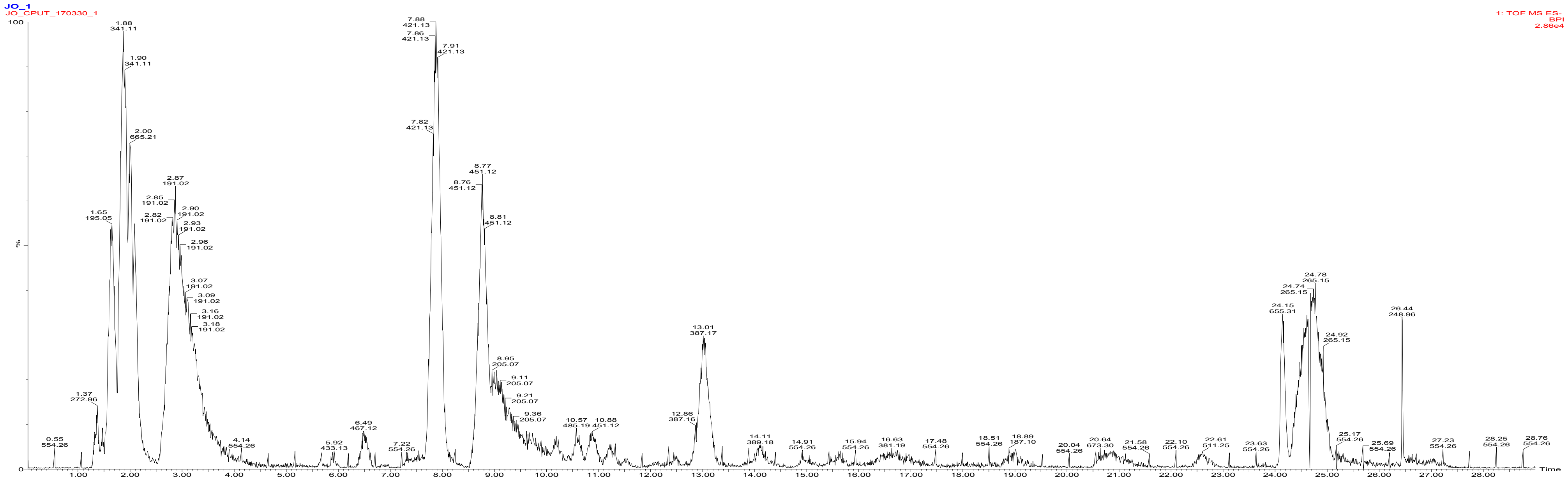

Figure S1. Chromatogram of phytochemicals and mass spectra of identified flavonoids in whole black Bambara groundnut variety.

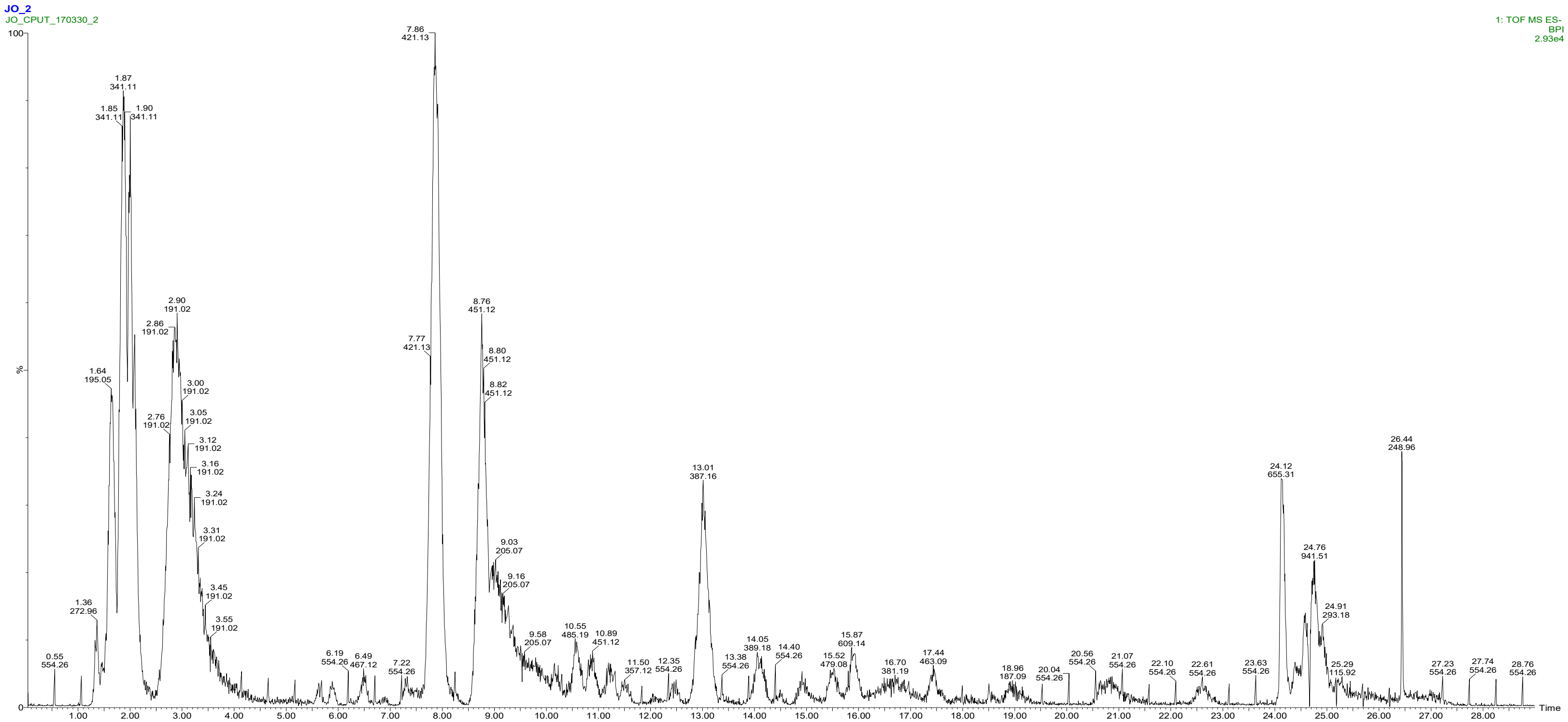

Figure S2. Chromatogram of phytochemicals and mass spectra of identified flavonoids in whole red Bambara groundnut variety.

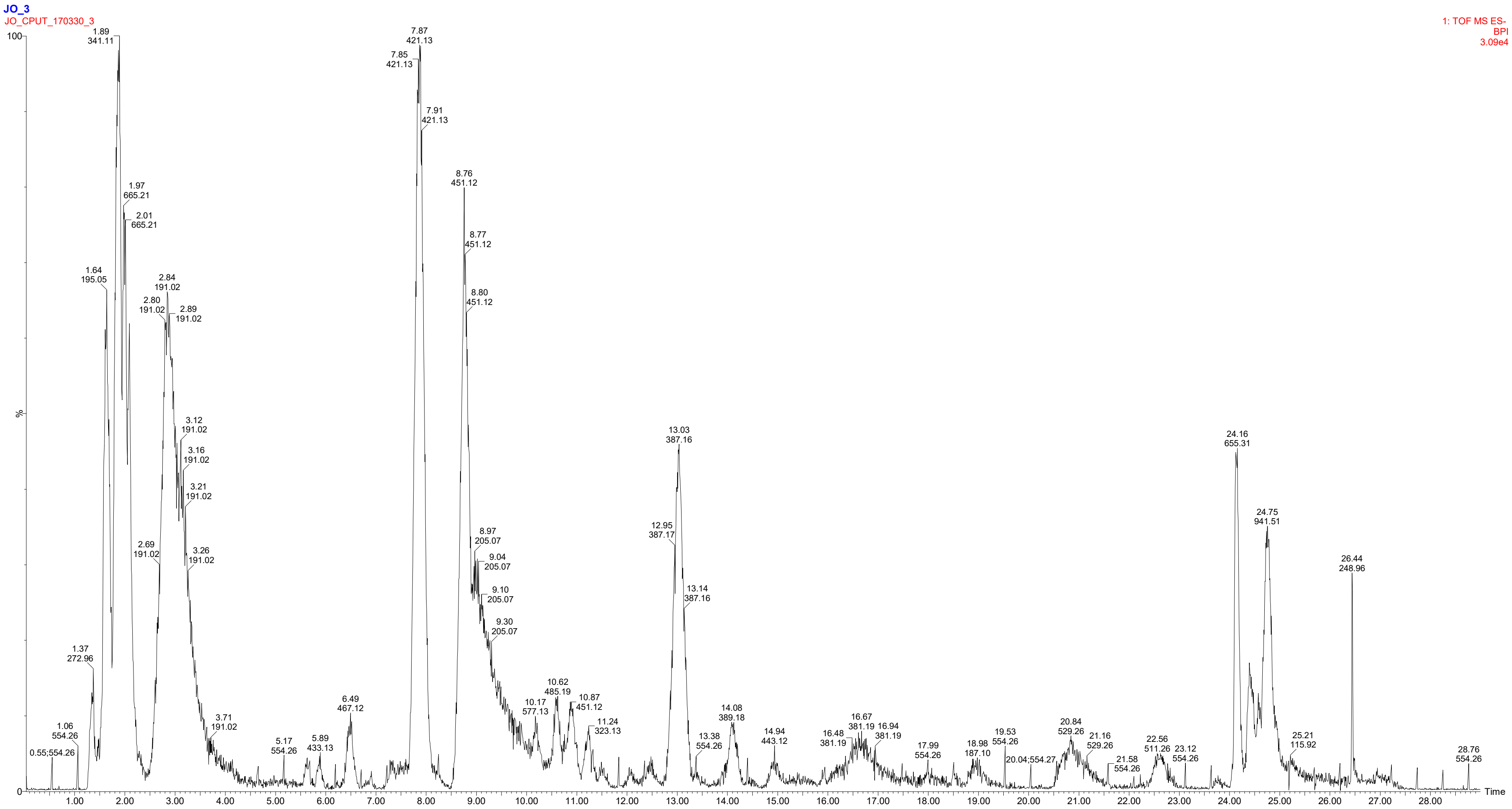

Figure S3. Chromatogram of phytochemicals and mass spectra of identified flavonoids in whole brown Bambara groundnut variety.

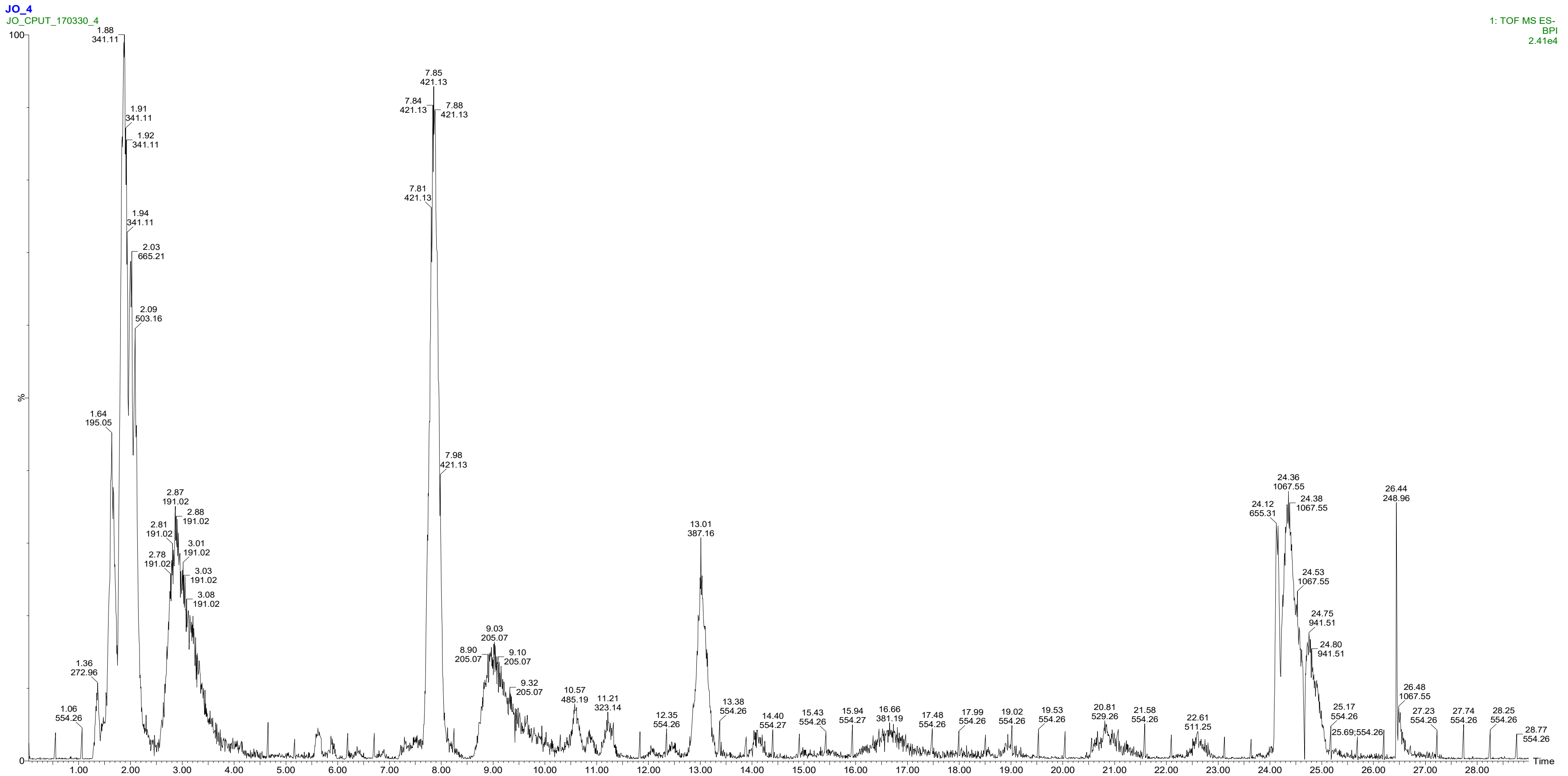

Figure S4. Chromatogram of phytochemicals and mass spectra of identified flavonoids in whole black-eyed Bambara groundnut variety.

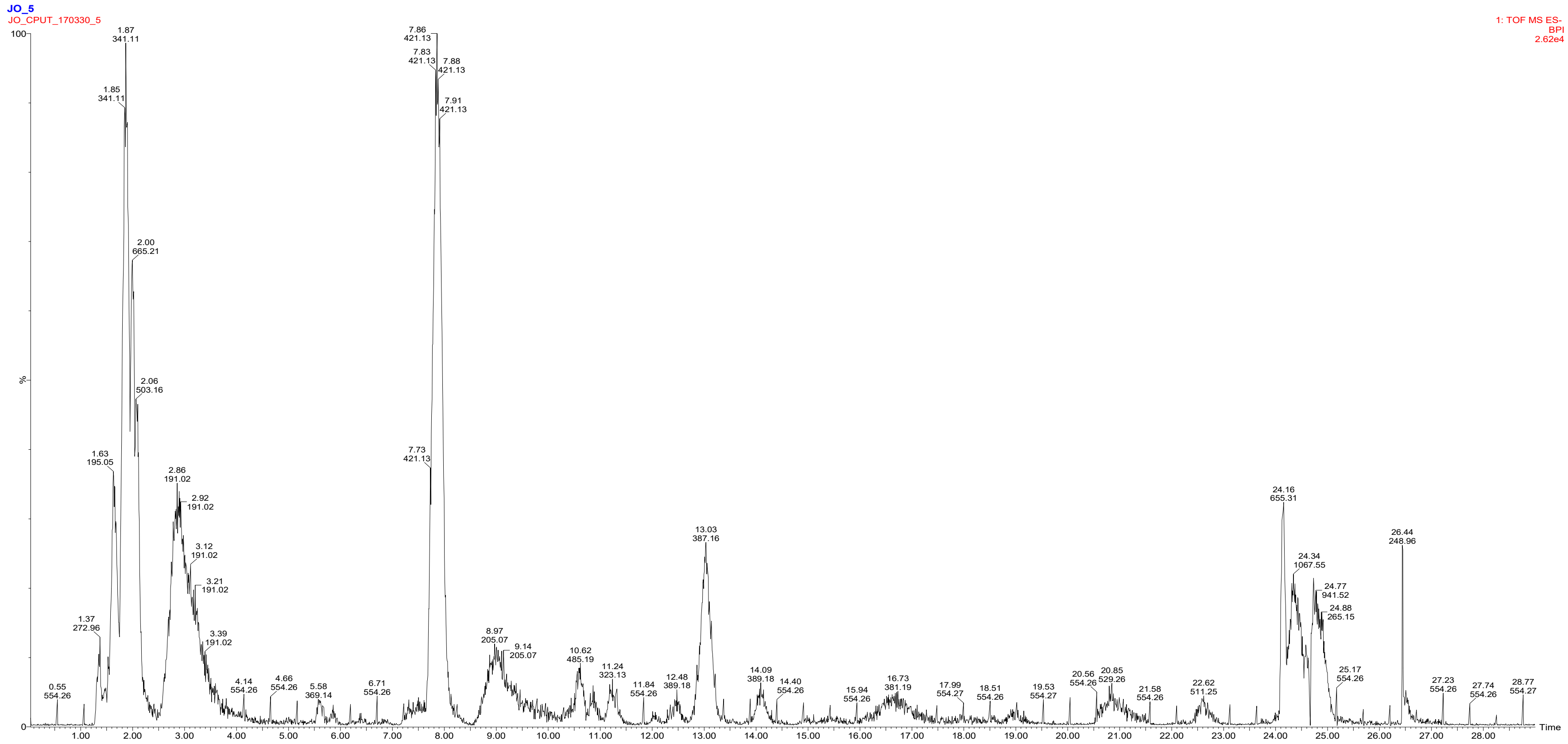

Figure S5. Chromatogram of phytochemicals and mass spectra of identified flavonoids in whole brown-eyed Bambara groundnut variety.

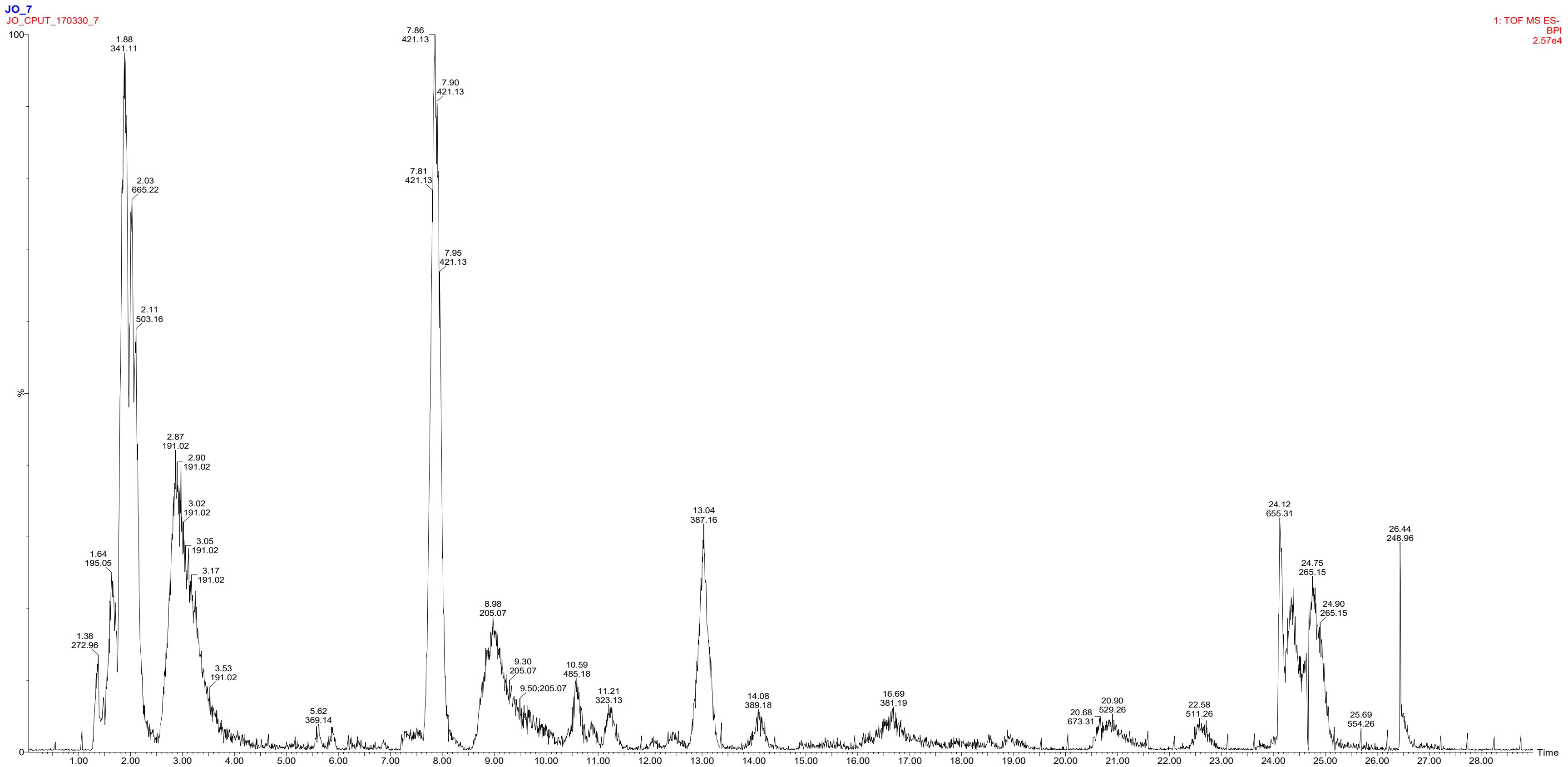

Figure S6. Chromatogram of phytochemicals and mass spectra of identified flavonoids in the cotyledon of black Bambara groundnut variety.

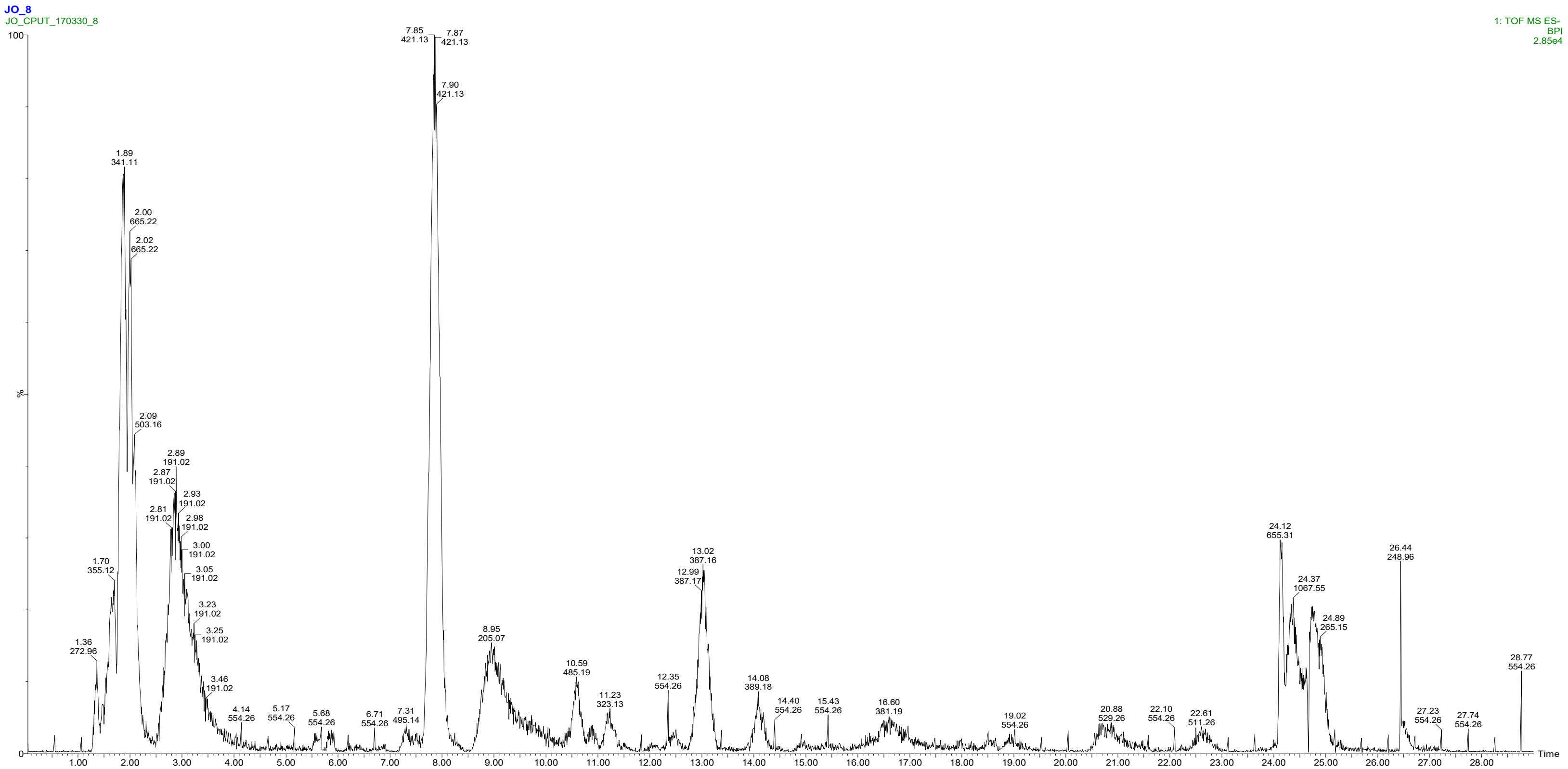

JO_8
JO_CPUT_170330_8
1: TOF MS ES-
BPI
2.85e4
100
%
0
7.85
421.13
7.87
421.13
7.90
421.13
1.89
341.11
2.00
665.22
2.02
665.22
2.09
503.16
2.89
191.02
2.87
191.02
2.93
191.02
2.81
191.02
2.98
191.02
3.00
191.02
3.05
191.02
3.23
191.02
3.25
191.02
3.46
191.02
1.70
355.12
1.36
272.96
4.14
554.26
5.17
554.26
5.68
554.26
6.71
554.26
7.31
495.14
8.95
205.07
10.59
485.19
11.23
323.13
12.35
554.26
13.02
387.16
12.99
387.17
14.08
389.18
14.40
554.26
15.43
554.26
16.60
381.19
19.02
554.26
20.88
529.26
22.10
554.26
22.61
511.26
24.12
655.31
24.37
1067.55
24.89
265.15
26.44
248.96
27.23
554.26
27.74
554.26
28.77
554.26
1.00
2.00
3.00
4.00
5.00
6.00
7.00
8.00
9.00
10.00
11.00
12.00
13.00
14.00
15.00
16.00
17.00
18.00
19.00
20.00
21.00
22.00
23.00
24.00
25.00
26.00
27.00
28.00
Time

Figure S7. Chromatogram of phytochemicals and mass spectra of identified flavonoids in the cotyledon of red Bambara groundnut variety.

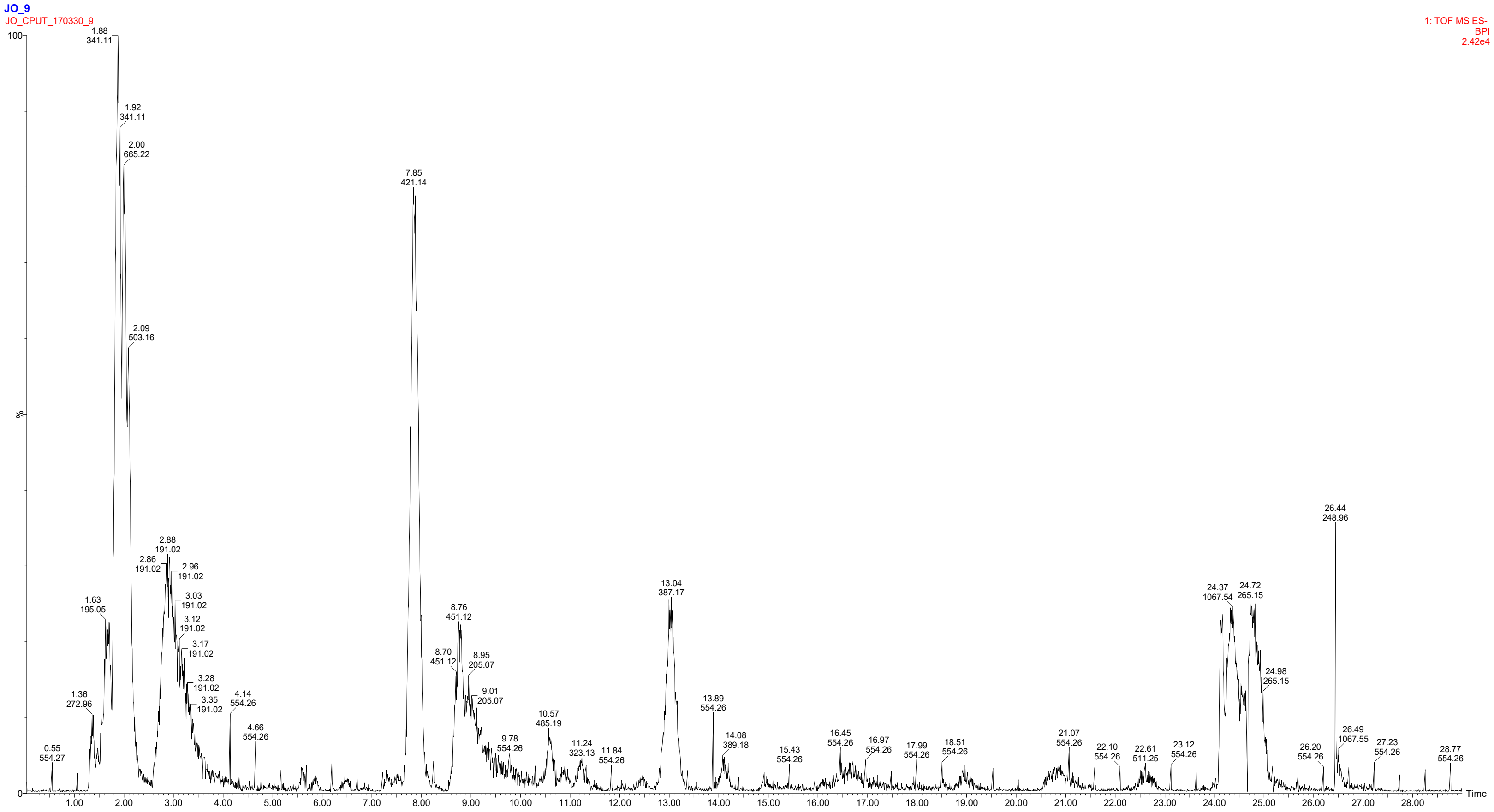

Figure S8. Chromatogram of phytochemicals and mass spectra of identified flavonoids in the cotyledon of brown Bambara groundnut variety.

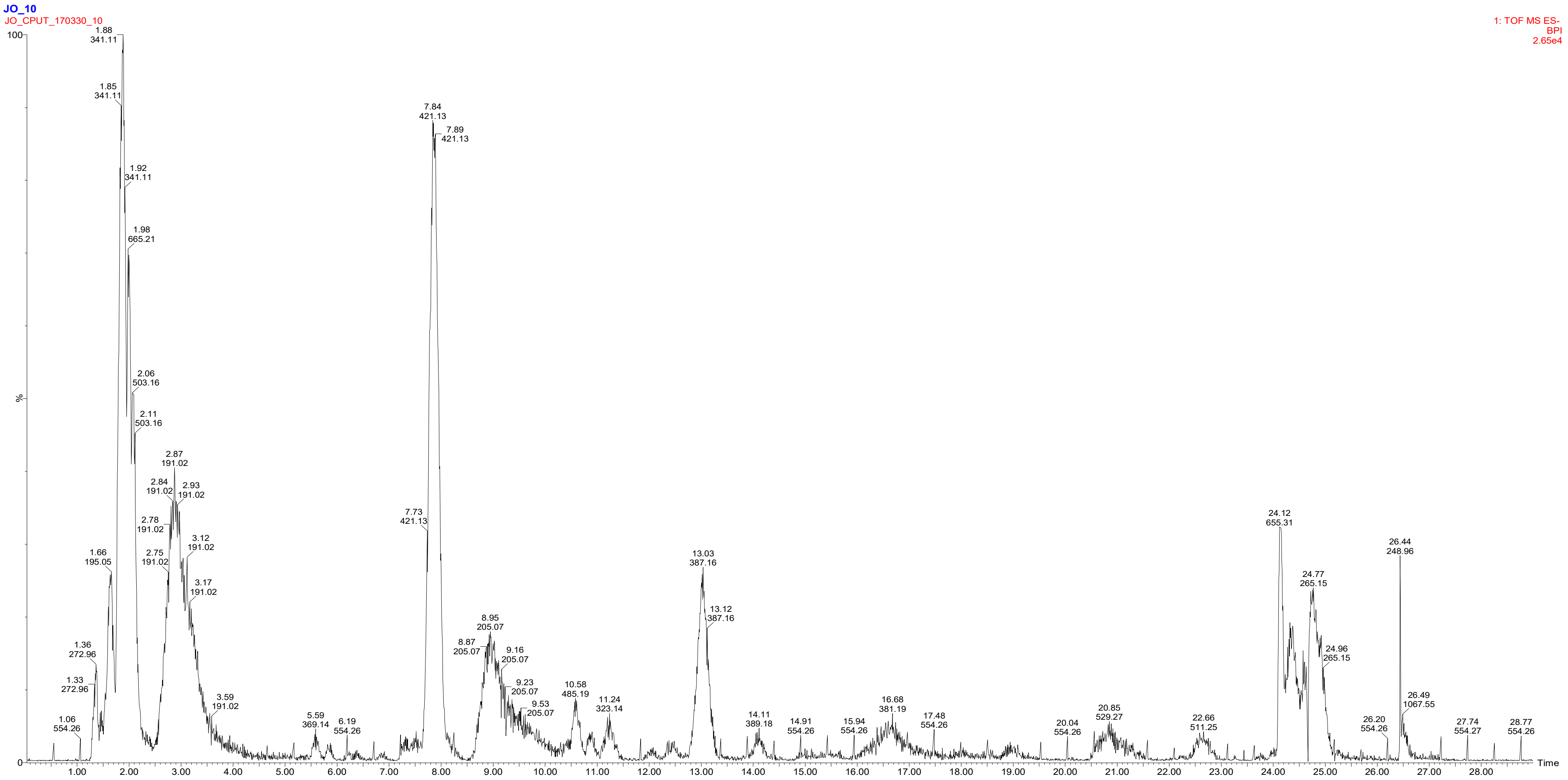

Figure S9. Chromatogram of phytochemicals and mass spectra of identified flavonoids in the cotyledon of black-eyed Bambara groundnut variety.

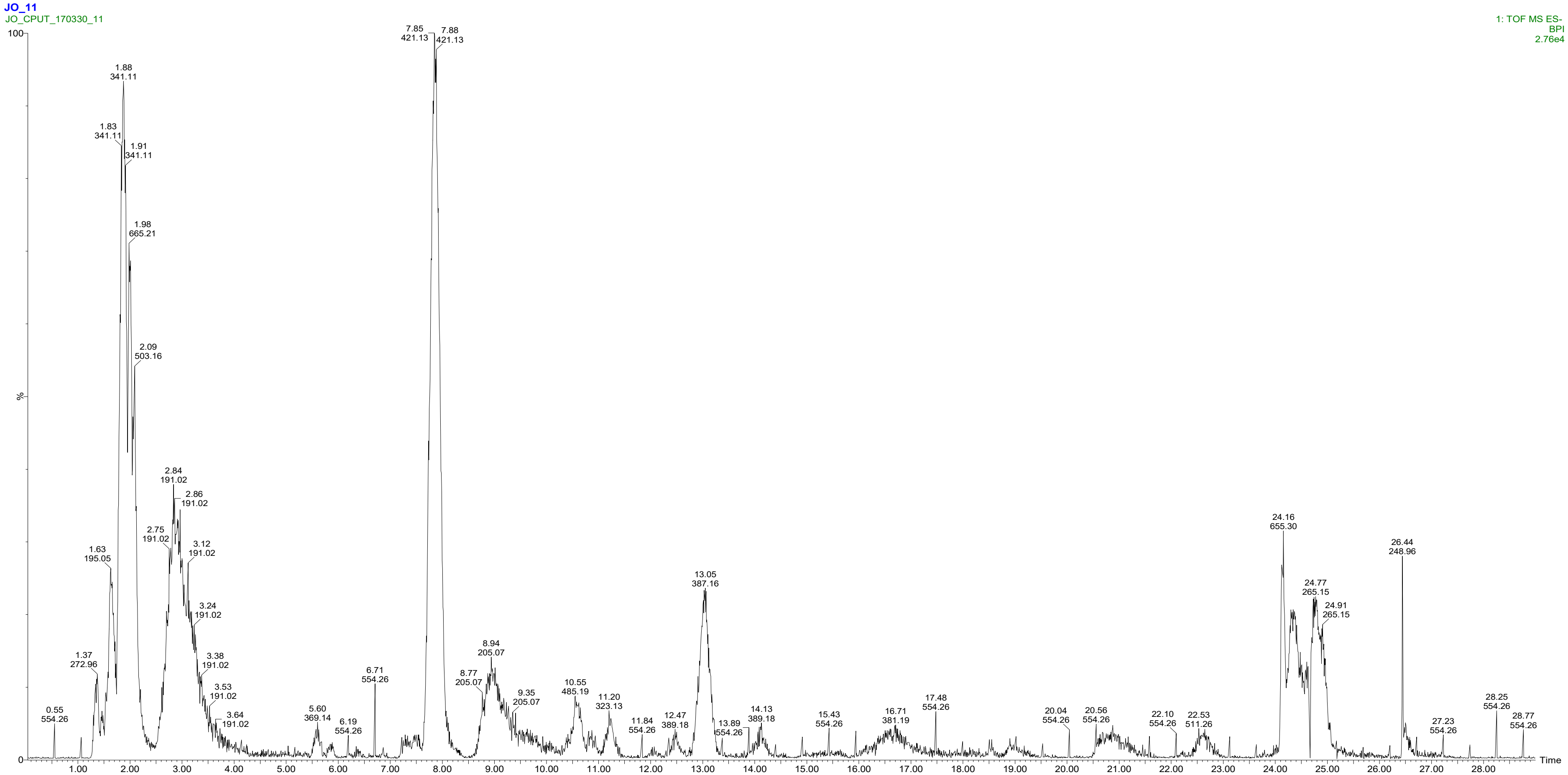

Figure S10. Chromatogram of phytochemicals and mass spectra of identified flavonoids in the cotyledon of brown-eyed Bambara groundnut variety.

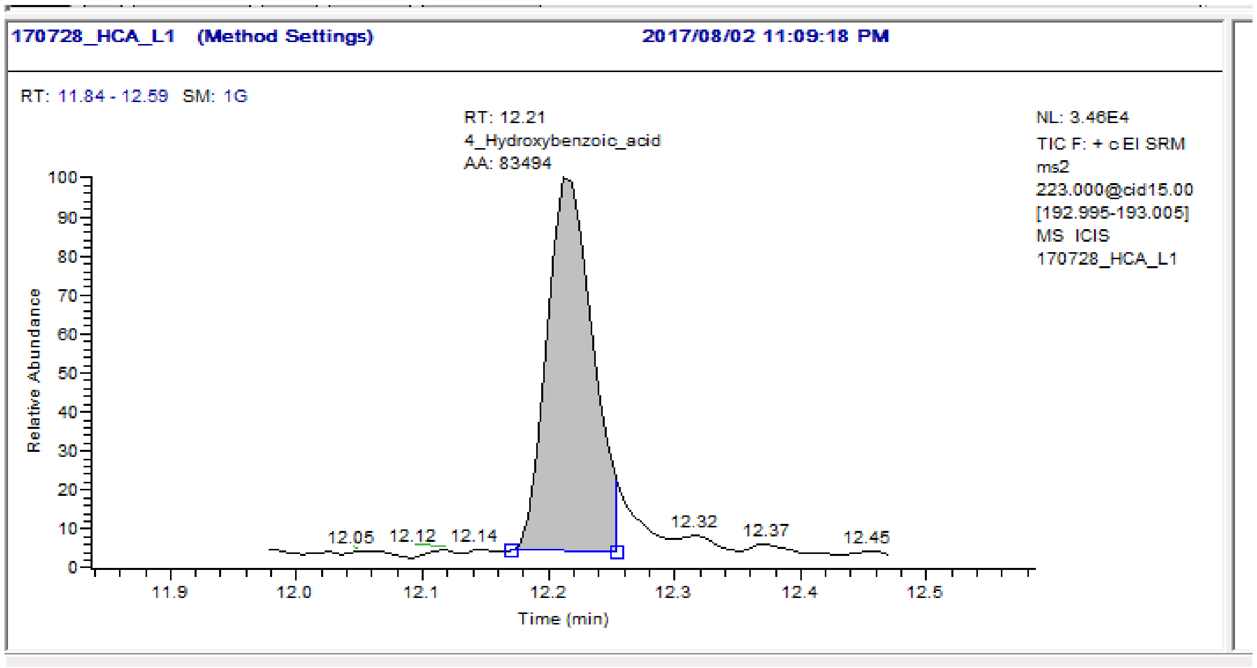

Figure S11. Chromatogram of phenolic acid and mass spectra of identified 4-Hydroxybenzoic acid Bambara groundnut varieties.

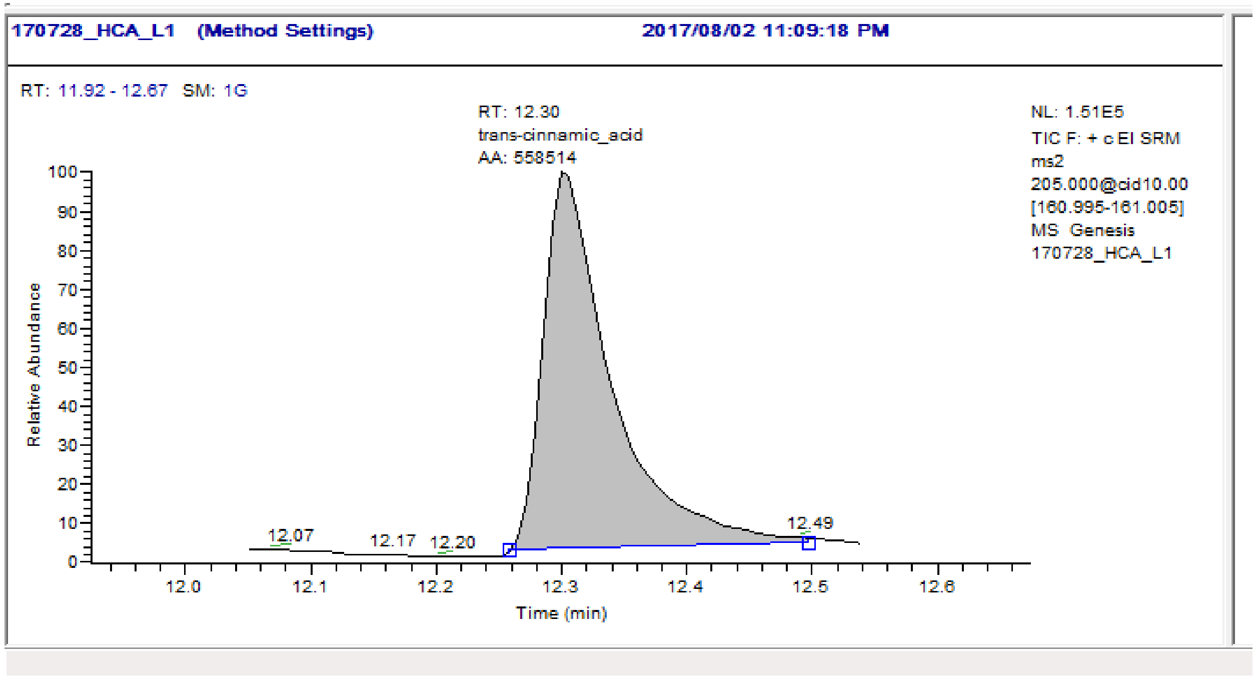

Figure S12. Chromatogram of phenolic acid and mass spectra of identified trans-cinnamic acid Bambara groundnut varieties.

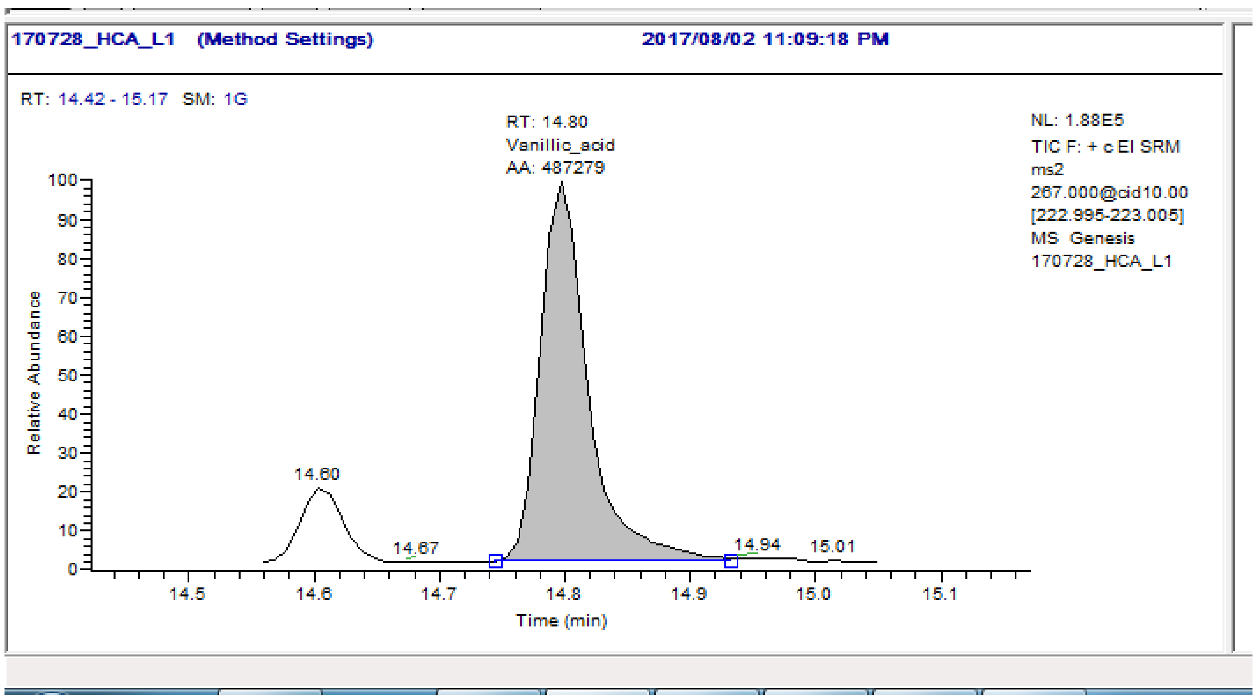

Figure S13. Chromatogram of phenolic acid and mass spectra of identified vanillic acid in Bambara groundnut varieties.

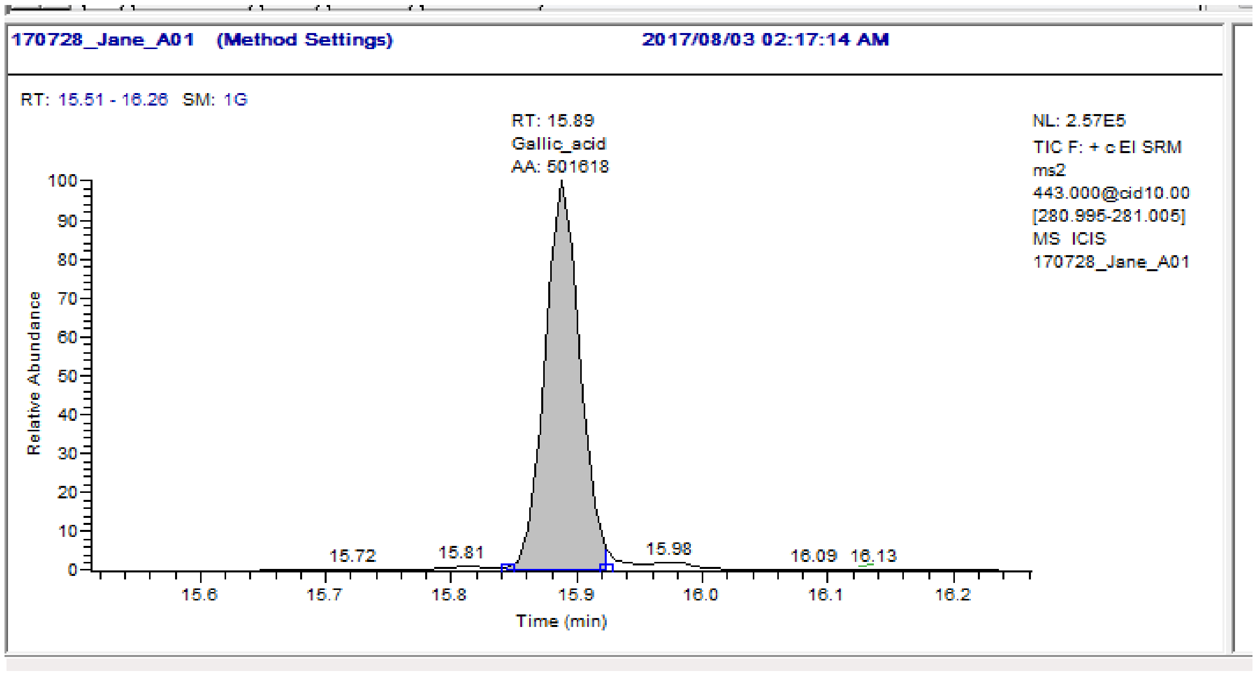

Figure S14. Chromatogram of phenolic acid and mass spectra of identified gallic acid in Bambara groundnut varieties.

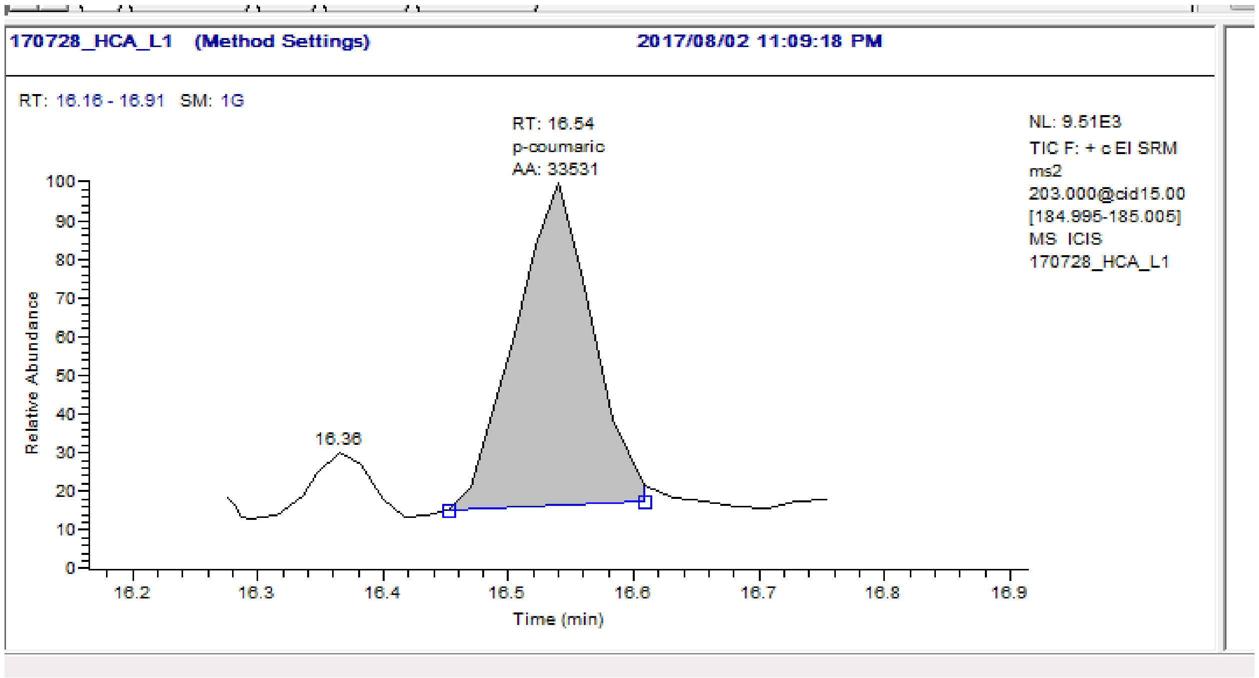

Figure S15. Chromatogram of phenolic acid and mass spectra of identified p-coumaric acid in Bambara groundnut varieties.

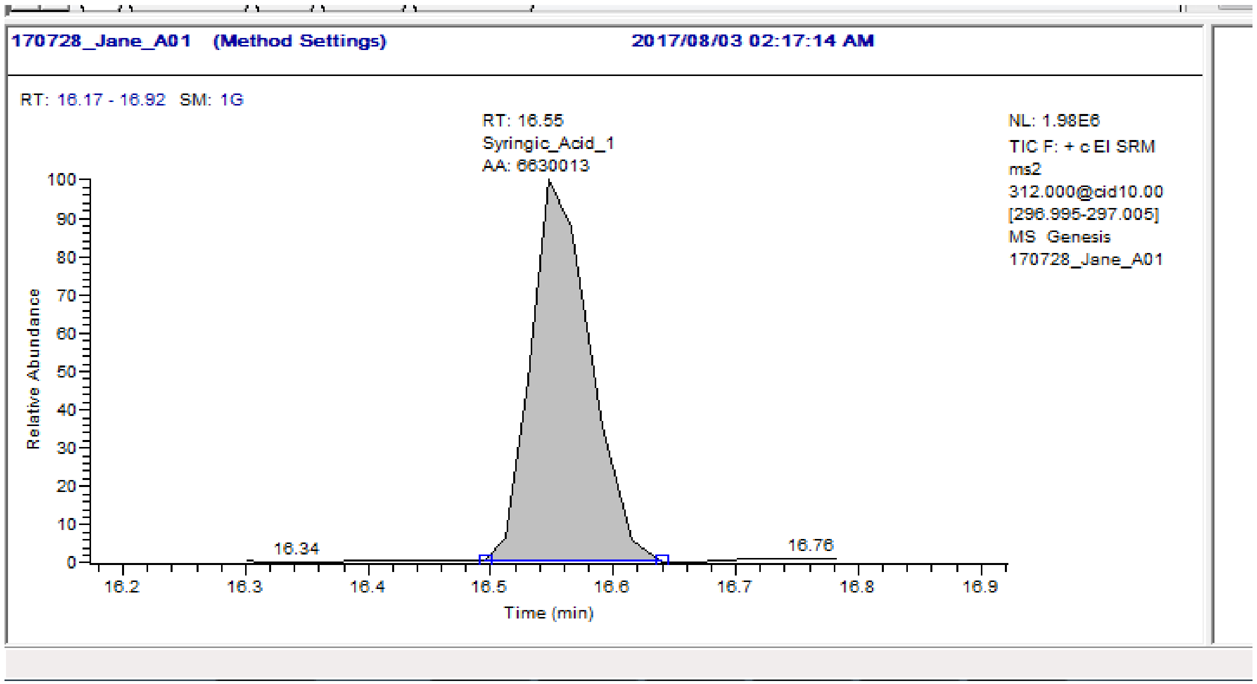

Figure S16. Chromatogram of phenolic acid and mass spectra of identified syringic acid Bambara in groundnut varieties.

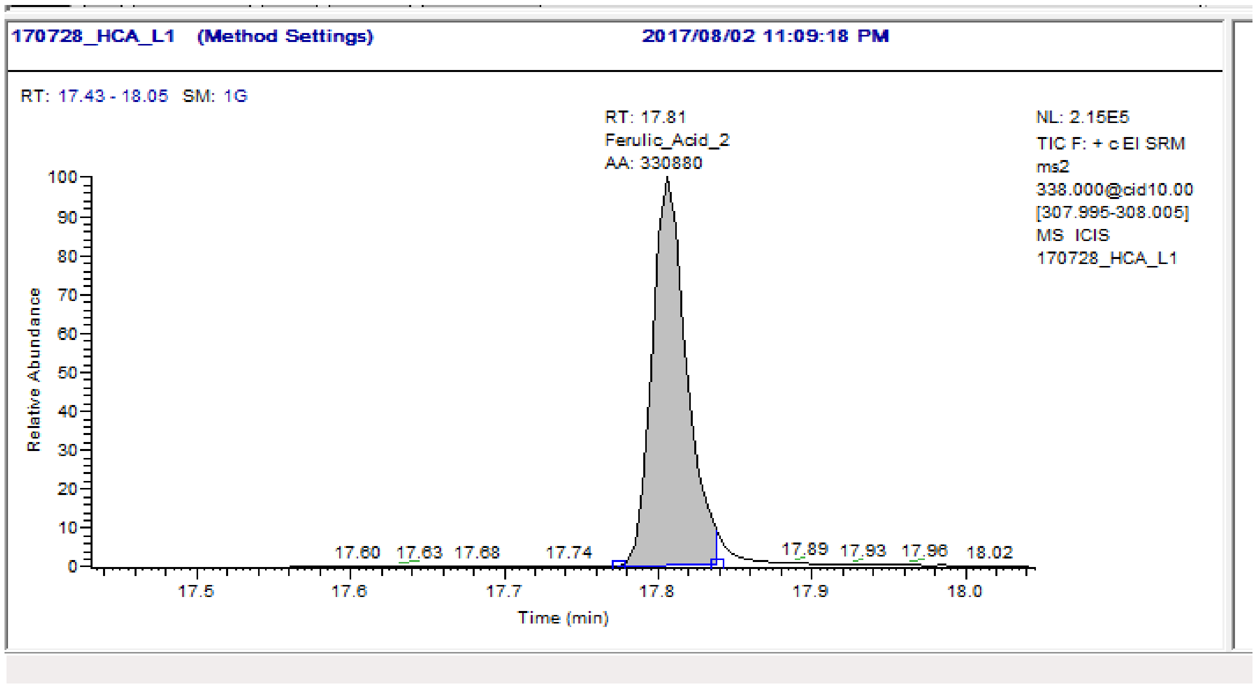

Figure S17. Chromatogram of phenolic acid and mass spectra of identified ferulic acid in Bambara groundnut varieties.
